# Tandem diaza-Cope rearrangement polymerization: turning intramolecular reaction into powerful polymerization to give enantiopure materials for Zn^2+^ sensors†

**DOI:** 10.1039/d0sc06138g

**Published:** 2020-12-08

**Authors:** Soon-Hyeok Hwang, Tae-Lim Choi

**Affiliations:** Department of Chemistry, Seoul National University Seoul 08826 Korea tlc@snu.ac.kr

## Abstract

[3,3]-Sigmatropic rearrangement is a powerful reaction to form C–C bonds stereospecifically; however, owing to intrinsic simultaneous bond formation and breakage, this versatile method has not been utilized in polymerization. Herein, we report a new tandem diaza-Cope rearrangement polymerization (DCRP) that can synthesize polymers with defect-free C–C bond formation from easy and efficient imine formation. A mechanistic investigation by *in situ*^1^H NMR experiments suggests that this polymerization proceeds by a rapid DCR process, forming an enantiospecific C–C bond that occurs almost simultaneously with imine formation. This polymerization produces not only highly stable polymers against hydrolysis due to resonance-assisted hydrogen bonds (RAHBs) but also chiral polymers containing enantiopure salen moieties, which lead to high-performance Zn^2+^-selective turn-on chemosensors with up to 73-fold amplification. We also found that their optical activities and sensing performances are heavily dependent on the reaction temperature, which significantly affects the stereoselectivity of DCR.

## Abstract 2

Herein, we report a new tandem diaza-Cope rearrangement polymerization synthesizing enantiopure polymers with defect-free C–C bond formation. Furthermore, these polymers can be applied as high-performance turn-on Zn^2+^ sensors.

## Graphical abstract

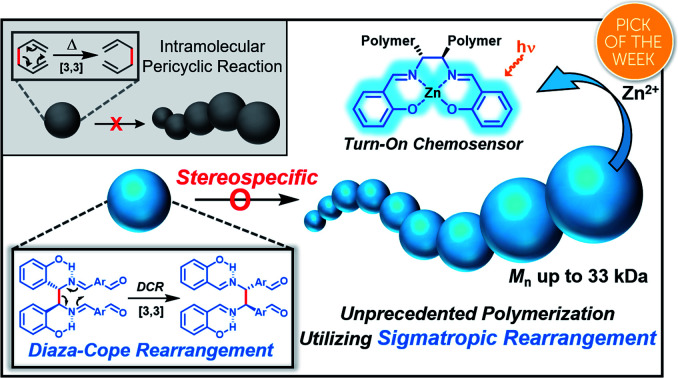

## Introduction

**Scheme 1:**
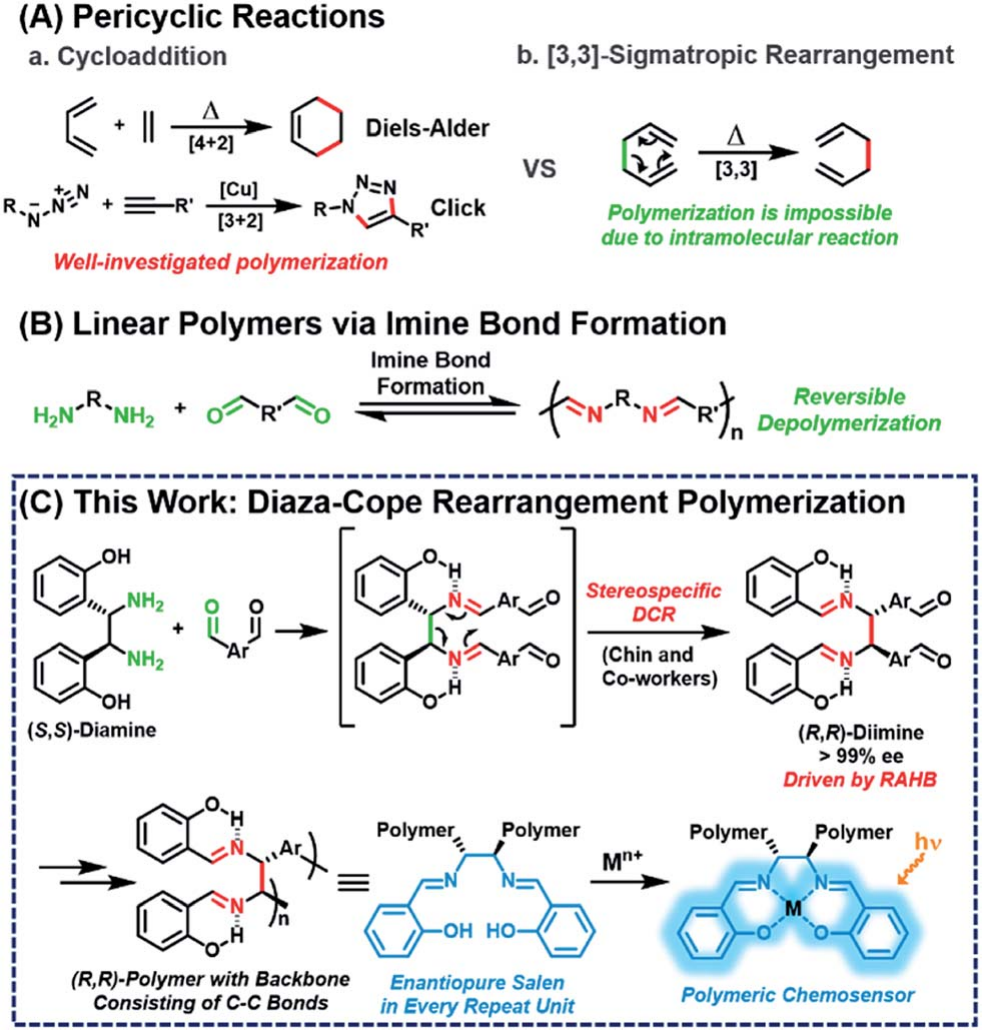
Polymerization *via* pericyclic reactions and imine formation.

Pericyclic reaction, the reorganization of π-bonds in a concerted manner, is one of the most widely used transformations in synthetic organic chemistry. Among the various pericyclic reactions, highly efficient cycloadditions, such as Diels–Alder and Cu-catalyzed azide–alkyne cycloaddition reactions, have been employed as powerful tools to prepare polymers^1–10^ because of their high efficiency and orthogonality (Scheme 1A(a)). [3,3]-Sigmatropic rearrangement is another powerful and reliable pericyclic reaction, allowing for the stereoselective construction of C–C bonds.^11^ However, this rearrangement cannot be applied to polymerization other than the post-modification of side-chains^12–16^ because it is an intramolecular reaction, which intrinsically forms and breaks the bond simultaneously (Scheme 1A(b)).

Meanwhile, imine formation between an amine and an aldehyde is an efficient and straightforward reaction and occurs reversibly under equilibrium control. Taking advantage of this dynamic nature, imine formation is widely employed for synthesizing polyimine vitrimers,^17–22^ rotaxanes, and catenanes.^23,24^ Ironically, extending this method to obtain high-molecular-weight linear polymers is still quite challenging due to its inherent reversible imine formation (Scheme 1B).^25–38^ Therefore, to solve the depolymerization issue, an appropriate strategy is needed to provide a sufficient driving force.

Then, we paid attention to Chin and co-workers' reports of highly versatile diaza-Cope rearrangement (DCR) reactions using chiral diamine and benzaldehydes.^39–47^ After efficient imine formation, DCR proceeded rapidly to form C–C bonds stereospecifically, driven by a strong resonance-assisted hydrogen bond (RAHB), thereby providing access to various enantiopure daughter amines. Inspired by this powerful DCR, we envisioned a new polymerization ironically using the intramolecular [3,3]-sigmatropic rearrangement in a tandem process with efficient imine formation. Herein, we report stereospecific and defect-free tandem diaza-Cope rearrangement polymerization (DCRP) between chiral diamine and bis-benzaldehyde *via* a rapid DCR process where the RAHB provided an enthalpic driving force (Scheme 1C). The DCR transforms the polymer backbone from reversible imine bonds to irreversible and strong C–C bonds; thus, this new polymerization provides high-molecular-weight linear polymers that are stable against hydrolysis. Furthermore, these chiral polymers containing salen side-chains can be applied as powerful turn-on chemosensors showing up to 73-fold amplification in fluorescence intensity.

## Results and discussion

To test the feasibility of DCRP, we screened various conditions using two commercially available diamine (*S*,*S*)-1 and bis-benzaldehyde 2a monomers with 10 mol% acid catalyst (*p*-TsOH) (Table 1). After testing several solvents at room temperature (rt), we found that the conversion increased (up to 96%) as the solvent polarity increased (entries 1–4). As a result, (*R*,*R*)-P1 with the highest number-average molecular weight (*M*_n_ = 14.9 kDa) was obtained in the most polar DMF (entry 4). Additionally, increasing the catalyst to 20 mol%, (*R*,*R*)-P1 with a higher *M*_n_ of 22.3 kDa and a reasonable *Đ* of 1.87 was obtained in 80% yield (entry 5 and Table S1†). Next, other bis-benzaldehydes (2b and 2c) were examined to expand the monomer scope. Likewise, (*R*,*R*)-P2 obtained from 2b also showed a high *M*_n_ of 33.3 kDa under identical conditions (entry 6 and Table S2†). Due to the solubility issue of the 2c monomer, the polymerization was carried out in chloroform at 40 °C to give (*R*,*R*)-P3 with a *M*_n_ of 16.7 kDa (entry 7 and Table S3†). Notably, the degree of polymerization (DP) calculated from the MALLS analysis matched the DP calculated from the Carothers equation using the conversion obtained by NMR analysis. Finally, all SEC traces showed a good Gaussian distribution, suggesting minimal cyclization (Fig. S6†).

**Table 1:** Optimization of tandem diaza-Cope rearrangement polymerization (DCRP)

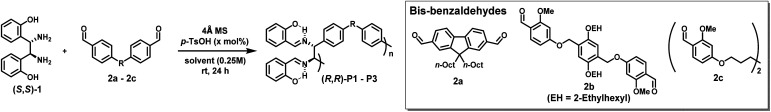							
Entry	Polymer	Bis-aldehyde	Solvent	*p*-TsOH (mol%)	Conv.^a^ (%)	*M*_n_ b (*Đ*)	Yield^c^ (%)
1	(*R*,*R*)-P1	2a	DCM	10	88	9.6 k (1.49)	97
2	(*R*,*R*)-P1	2a	CHCl_3_	10	88	9.2 k (1.53)	91
3	(*R*,*R*)-P1	2a	THF	10	93	14.6 k (1.63)	76
4	(*R*,*R*)-P1	2a	DMF	10	96	14.9 k (1.40)	64
5	(*R*,*R*)-P1	2a	DMF	20	97	22.3 k (1.87)	80
6	(*R*,*R*)-P2	2b	DMF	20	97	33.3 k (1.61)	90
7^d^	(*R*,*R*)-P3	2c	CHCl_3_	20	96	16.7 k (1.76)	78
8^e^	(*S*,*S*)-P1	2a	DMF	20	97	20.1 k (2.34)	98
9^f^	*meso*-P1	2a	DMF	20	83	9.4 k (1.60)	71

a Determined by ^1^H NMR analysis of the crude reaction mixture.

b Absolute molecular weights determined by THF SEC using a multiangle laser light scattering (MALLS) detector.

c Isolated yields after purification from isopropyl alcohol (IPA).

d Polymerization proceeded in chloroform at 40 °C to enhance monomer solubility.

e (*R*,*R*)-1 was employed instead of (*S*,*S*)-1.

f Polymerization proceeded using *meso*-1 instead of (*S*,*S*)-1 at 50 °C.

**Fig. 1:**
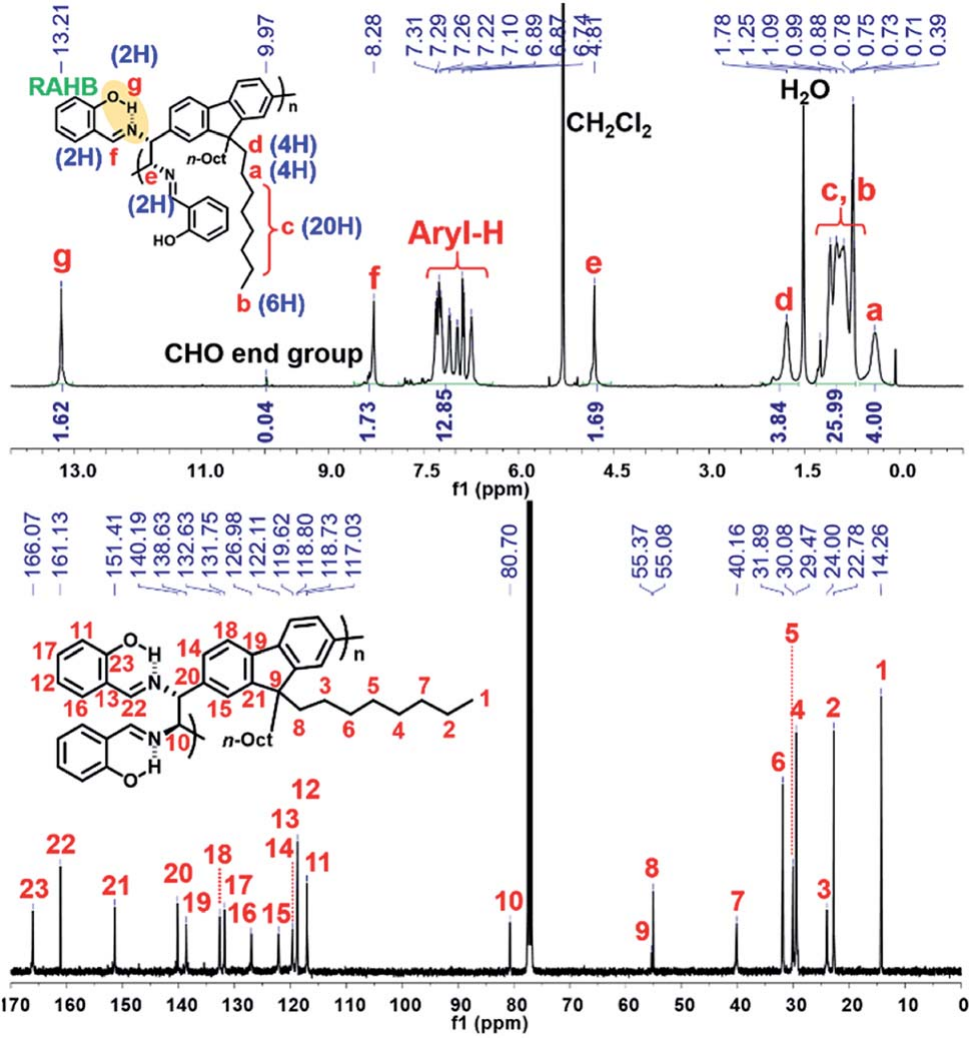
^1^H NMR and ^13^C NMR spectra of (*R*,*R*)-P1.

The detailed microstructures of all resulting polymers were easily characterized by ^1^H NMR and ^13^C NMR analysis due to the sharp and well-resolved NMR spectra, facilitating easy assignment of all peaks (Fig. 1 for (*R*,*R*)-P1). Notably, the phenolic O–H proton signals (g peak for (*R*,*R*)-P1) of all the resulting polymers commonly appeared at *δ* 13.2–13.8 ppm as sharp singlets. Considering that the phenolic O–H signals formed by normal hydrogen-bonding are generally observed at *ca. δ* 11 ppm, which is completely absent in Fig. 1, these new peaks at *ca. δ* 14 ppm are significantly downfield-shifted due to the unique RAHB.^42^ This observation confirms that the DCR process successfully occurred during the polymerization without any defect.

**Fig. 2:**
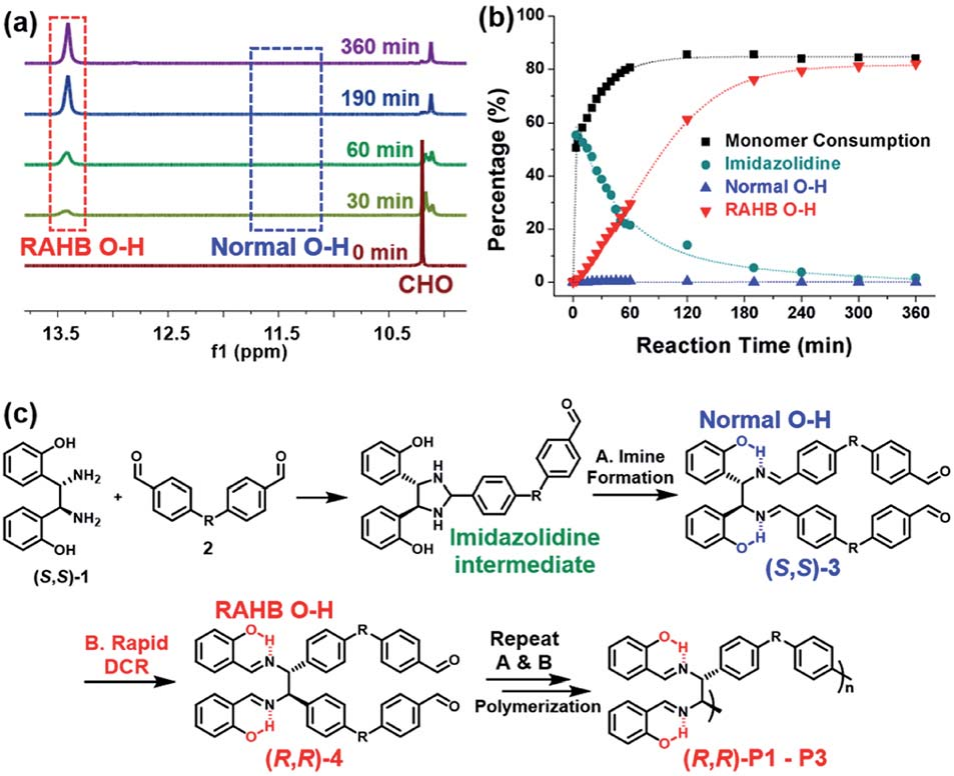
(a) Monitoring the polymerization using (*S*,*S*)-1 and 2a by *in situ*^1^H NMR spectroscopy in DMF-*d*_7_. (b) Plots showing consumption of 2a (black), formation of the imidazolidine intermediate (green), and phenolic O–H groups (blue and red). (c) Detailed polymerization mechanism.

To get a mechanistic insight into tandem DCRP, the polymerization using (*S*,*S*)-1 and 2a was monitored by *in situ*^1^H NMR experiments under the same conditions as entry 5 in Table 1 (Fig. 2 and S1†). As reported, an initial imidazolidine intermediate *via* imine formation was rapidly formed up to 55% (see Fig. S1† for details).^46,47^ However, (*S*,*S*)-3, the second intermediate, comprising the normal O–H signal, was hardly observed, whereas only the RAHB O–H signal corresponding to (*R*,*R*)-4 or (*R*,*R*)-P1 gradually increased, implying clean conversion (Fig. 2a and b). This suggests that a DCR is rapid, occurring almost simultaneously with imine formation (Fig. 2c). In other words, (*R*,*R*)-P1 was predominantly produced by C–C bond polymerization rather than imine polymerization (if so, the subsequent DCR process would have slowly generated C–C bonds on the pre-formed polymer). On the other hand, in the case of (*R*,*R*)-P2 containing electron-donating groups, the activation energy for DCR increased,^42^ thereby decelerating the rearrangement. As a result, both normal and RAHB O–H (*δ* 10.7 ppm and *δ* 13.7 ppm, respectively) were observed at the beginning of the reaction, but the polymerization progressed to show only the enthalpically favored RAHB O–H (Fig. S2†). In short, because of the defect-free DCR signal regardless of the electronic characters, this tandem DCRP becomes a novel strategy to form more challenging C–C bonds from easier imine formation.

**Fig. 3:**
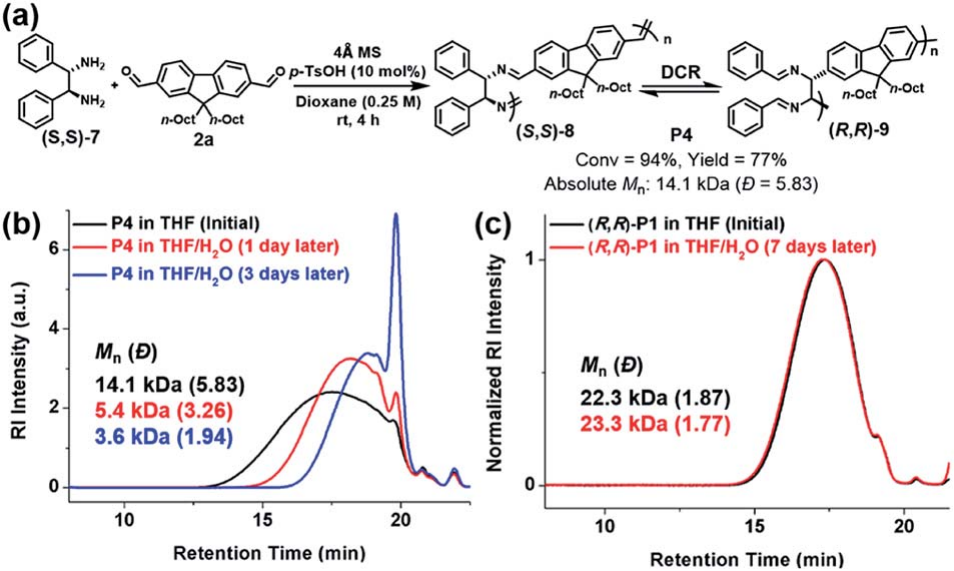
Control experiment to compare stability against hydrolysis. (a) Scheme for P4. (b) SEC traces of P4 measured at the initial and measured one/three days after dissolving in THF : H_2_O (99 : 1). (c) SEC traces of (*R*,*R*)-P1 measured at the initial and seven days after dissolving in THF : H_2_O (99 : 1).

DCR polymers contain C–C bonds in the main backbone, while the resulting imine bonds at the side-chains are also stabilized by stronger RAHBs. Therefore, we could test their stability against hydrolysis by comparing with analogous polyimine. To conduct a control experiment, we synthesized P4 by using a diamine monomer ((*S*,*S*)-7) without –OH groups, ensuring that the RAHB that drove the completion of DCR was now absent.^39^ Therefore, P4 (*M*_n_ of 14.1 kDa) contained both the initial imine ((*S*,*S*)-8) and the rearranged C–C bond ((*R*,*R*)-9) in the main chain (Fig. 3a). As expected, P4 in the THF solution containing 1% H_2_O underwent depolymerization, lowering *M*_n_ to 5.4 kDa within a day. After three days, P4 was further hydrolyzed to 3.6 kDa, which is 1/4 of the initial molecular weight (Fig. 3b). However, as shown by SEC analysis, the molecular weight of (*R*,*R*)-P1 from the complete DCRP did not change under identical conditions even after seven days (Fig. 3c). Moreover, (*R*,*R*)-P1 remained stable in CDCl_3_ in an NMR tube for three days, but even a small amount of residual acid in CDCl_3_ was sufficient to depolymerize P4 within 11 h, as seen by the reduction in the imine signal (*δ* 8.3 ppm) and the significant increase in the aldehyde signal (*δ* 10.0 ppm) (Fig. S3†).

**Fig. 4:**
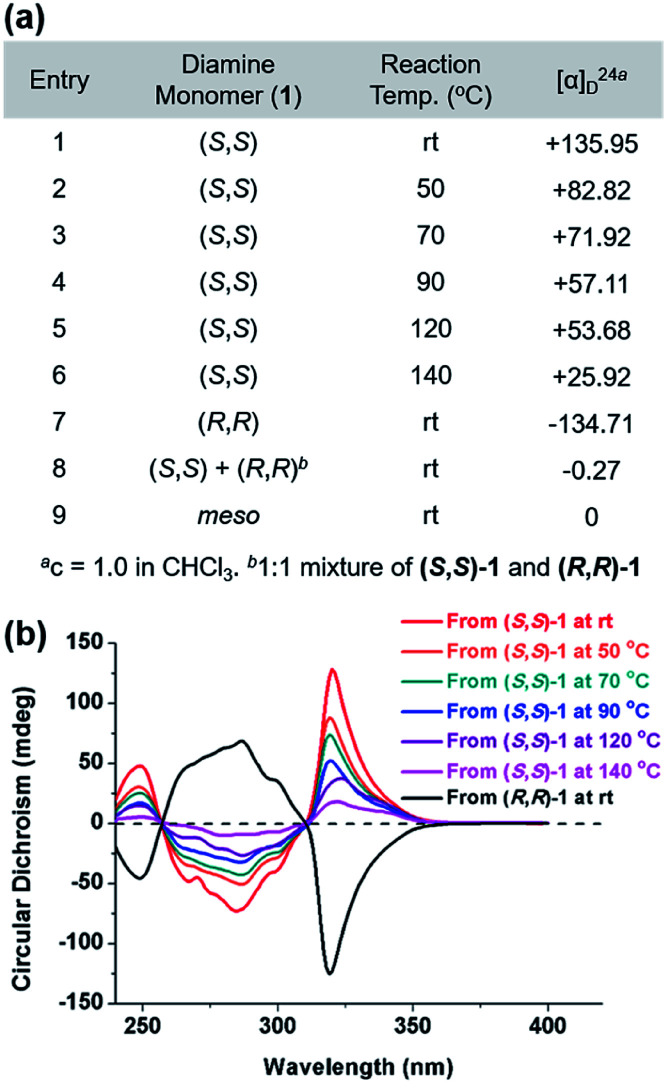
(a) Table for optical rotation values showing how temperature affects the stereospecificity of tandem DCRP. (b) Circular dichroism spectra of the resulting polymers (2.5 mg mL^−1^ in CHCl_3_ at rt, 0.2 mm cell).

The DCR of small molecules undergoes complete stereospecific inversion of stereochemistry at rt because the corresponding transition state (TS) bears all the aryl substituents in pseudo-equatorial positions making this pathway most kinetically preferred.^42^ Likewise, to investigate the stereochemistry of the resulting polymers, we prepared P1 at various temperatures and compared their optical rotations ([*α*]^24^_D_) (Fig. 4a and Table S4†). While P1 synthesized from (*S*,*S*)-1 at rt exhibited the highest [*α*]^24^_D_ of +135.95, the values significantly decreased from +82.82 to +25.92 as the polymerization temperature increased from 50 °C to 140 °C (entries 1–6 in Fig. 4a). In addition, (*S*,*S*)-P1 obtained from the enantiomer (*R*,*R*)-1 at rt (entry 8 in Table 1) showed an expected [*α*]^24^_D_ of similar absolute value but the opposite sign (−134.71) (entry 7 in Fig. 4a). Lastly, P1 prepared from the racemic mixture or *meso*-isomer of 1 resulted in [*α*]^24^_D_ close to 0 (entries 8 and 9 in Fig. 4a). To further support this trend, we measured circular dichroism spectra, which showed a decrease in the amplitudes of the two Cotton effects with an increase in the reaction temperature (Fig. 4b). Furthermore, (*R*,*R*)-P1 and (*S*,*S*)-P1 synthesized at rt showed Cotton effects of the same amplitude but of the opposite sign. According to the computational studies, the next alternating pathway *via* the second lowest TS (7.7 kcal mol^−1^ higher) would produce a *meso* product, an achiral diastereomer.^42^ Therefore, it is suspected that the higher reaction temperature led to more *meso* products in P1, thereby lowering [*α*]^24^_D_ and the amplitude of Cotton effects accordingly.

**Fig. 5:**
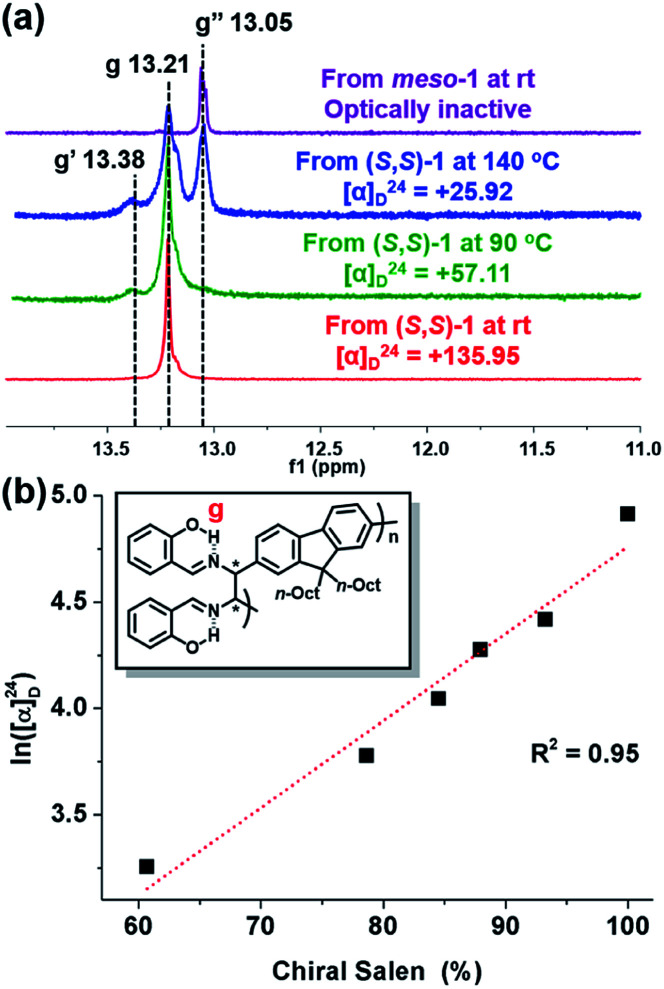
(a) ^1^H NMR spectra (in CD_2_Cl_2_) showing RAHB O–H signals of various P1 (in Table S4†) synthesized from chiral and *meso* diamines at various reaction temperatures. (b) Plot showing the relationship between ln([*α*]^24^_D_) and stereospecificity.

To confirm the effect of temperature on *meso*-isomer formation, we thoroughly analyzed the ^1^H-NMR spectra of P1 from (*S*,*S*)-1 and *meso*-P1 synthesized at various temperatures (Fig. 5a and S4†). As the polymerization temperature increased, another peak labeled as g′ appeared and gradually increased at *δ* 13.38 ppm (Fig. 5a and S4a–f†). Notably, apart from the g and g′ peaks, an additional RAHB O–H signal (g′′ at *δ* 13.05 ppm) started to grow for P1 synthesized at higher temperatures. Fortunately, the last g′′ peak was easily identified as the *meso* diastereomer as it perfectly matched with the authentic sample of *meso*-P1 prepared from *meso*-1 at rt (Fig. 5a and S4g†). From these data, we came to a plausible conclusion that the three types of RAHB O–H signals are due to diad tacticities of the salen side-chains in the following cases: (i) g for the homo diad (cm^3^) from the chiral-salen-rich units; (ii) g′′ for the homo diad (mm) from the achiral *meso*-salen-rich units; (iii) g′ for the hetero diad (cm) from the chiral and *meso*-salen-rich units adjacent to each other. Based on this assignment, the content of the chiral salen unit depending on the temperature was estimated from the integration of all RAHB O–H signals (Fig. S5a†). Consistent with the trend observed in [*α*]^24^_D_, the chiral salen content also decreased from 100% to 60.7% as the temperature increased from rt to 140 °C. Moreover, one could plot [*α*]^24^_D_ to the chiral content, and they showed an exponential relationship (Fig. S5b†). Taking the natural logarithm of [*α*]^24^_D_ provided a good linear relationship with the chiral content, which should allow for some predictability (Fig. 5b). In short, increasing the reaction temperature lowered the stereospecificity as well as [*α*]^24^_D_ due to the formation of the *meso*-isomer.

**Fig. 6:**
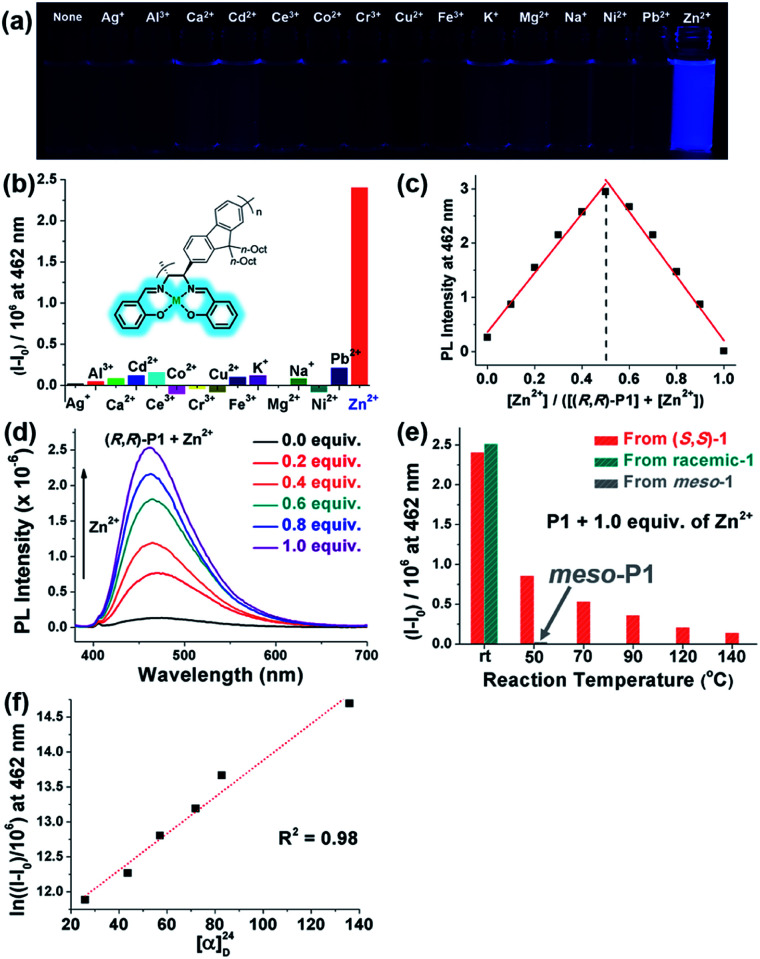
Metal sensing experiments using P1 in THF : H_2_O = 9 : 1 (v/v) solution (10 μM) at 298 K. (a) Photoluminescence of (*R*,*R*)-P1 solution in the absence or presence of 1.0 equiv. of various metal cations under 365 nm UV irradiation. (b) Emission changes of (*R*,*R*)-P1 in the presence of 1.0 equiv. of various metal cations. (c) Job plot of (*R*,*R*)-P1*vs.* Zn^2+^ (total concentration = 20 μM). (d) Emission spectra of (*R*,*R*)-P1 with increasing amounts of Zn^2+^ (0.0–1.0 equiv.). (e) Effect of polymerization temperature on the Zn^2+^ sensing performance of P1. (f) Correlation between fluorescence enhancement (*I* − *I*_0_) and optical rotation ([*α*]^24^_D_) of P1.

One useful application of salen moieties is their potential as a metal chemosensor.^48–50^ Since (*R*,*R*)-P1–P3 contain salen side-chains, their UV/vis absorption and fluorescence responses to various metal cations in THF/H_2_O (9 : 1 v/v) were investigated (Fig. 6 and S8–S12†). Interestingly, all the metal cations showed insignificant fluorescence except for Zn^2+^, which showed obvious turn-on fluorescence enhancement (*I* − *I*_0_) (Fig. 6a, b and S8†). On the other hand, no sensing ability was observed for P4, which only contains diimine side-chains without –OH groups (Fig. S9†). The Job plot using (*R*,*R*)-P1 showed the maximum fluorescence intensity at 0.5 mole fraction, indicating 1 : 1 complexation of Zn^2+^ to the salen group (Fig. 6c). Increasing the concentration of Zn^2+^ from 0.0 to 1.0 equiv. resulted in a gradual increase in the fluorescence intensities. In particular, (*R*,*R*)-P3 was an excellent Zn^2+^ sensor showing a high amplification of fluorescence (*I*/*I*_0_) up to 73-fold and a limit of detection value of 0.577 μM (37.7 ppb) (Fig. 6d, S10 and S11†). Notably, P1 prepared at various temperatures showed different sensing performances (Fig. 5e and S12†). P1 synthesized at rt using enantiopure (*S*,*S*)-1 or the racemic mixture of chiral 1 exhibited the highest *I*/*I*_0_ of 19-fold, whereas this *I*/*I*_0_ decreased significantly with the temperature to 4.8-fold (25% compared to that at rt) at 50 °C, 2.6-fold (14%) at 90 °C, 1.9-fold (10%) at 120 °C, and even further down to 1.5-fold (8%) at 140 °C. The *I* − *I*_0_ and [*α*]^24^_D_ also showed an exponential relationship and applying the natural logarithm to *I* − *I*_0_ provided a good linear relationship with [*α*]^24^_D_, allowing for prediction of the sensor performances (Fig. 6f and S12c†). Furthermore, to examine the effect of the *meso*-salen unit, we conducted a sensing experiment using *meso*-P1 prepared at 50 °C to achieve comparable conversion (entry 9 in Table 1 and Fig. S4h†). Interestingly, *meso*-P1 showed no sensing ability for Zn^2+^ at all, indicating that the stereochemistry of the salen moiety was crucial for Zn^2+^ detection (Fig. 6e). Therefore, we concluded that the high sensing performances of (*R*,*R*)-P1–P3 synthesized at rt were due to the exclusive formation of the chiral salens, which formed a tetrahedral geometry suitable for selective Zn^2+^ binding.^50^ However, as the reaction temperature increased, the degree of DCR to the undesired *meso*-isomer increased, and their sensing performance decreased due to the resulting square-planar geometry from the *meso* configuration in which selective Zn^2+^ detection is impossible.

## Conclusions

In conclusion, we developed a new tandem diaza-Cope rearrangement polymerization (DCRP) to synthesize high-molecular-weight polymers up to 33.3 kDa with excellent stability against hydrolysis. The key factor for the success was the introduction of a defect-free, rapid, and thermodynamically favored DCR process into the polymerization mechanism by forming C–C bonds almost simultaneously with efficient imine formation. Taking advantage of the highly stereospecific DCR, we prepared several highly enantiopure polymers having either (*R*,*R*)- or (*S*,*S*)-salen moieties. Lastly, these polymers showed excellent performance as turn-on chemosensors selective for Zn^2+^ detection.

## Conflicts of interest

There are no conflicts to declare.

## Supplementary Material

SC-012-D0SC06138G-s001
