# Supplementary material for: Tandem diaza-Cope rearrangement polymerization: turning intramolecular reaction into powerful polymerization to give enantiopure materials for Zn2+ sensors
Source: Chem Sci. 2020 Dec 8;12(7):2404–9. doi: 10.1039/d0sc06138g (PMC8179250; doi:10.1039/d0sc06138g)
Supplement: SC-012-D0SC06138G-s001 [file SC-012-D0SC06138G-s001.pdf]

Electronic Supplementary Material (ESI) for Chemical Science.
This journal is © The Royal Society of Chemistry 2020

Supporting Information

Tandem Diaza-Cope Rearrangement Polymerization: Turning Intramolecular Reaction into Powerful Polymerization to Give Enantiopure Materials for Zn^{2+} Sensor

Soon-Hyeok Hwang and Tae-Lim Choi*
Department of Chemistry, Seoul National University, Seoul 08826, Korea.

*E-mail: tlc@snu.ac.kr

Table of Contents

I. General Methods ····· S2

II. Preparation and Characterization of Monomers ····· S2

III. General Procedure for the Polymerization and Characterization of Polymers ····· S3

IV. Optimization of Polymerization ····· S5

V. *In situ* ^{1}NMR Experiment in DMF-d_7 ····· S6

VI. Control Experiment to Compare Stability against Hydrolysis ····· S7

VII. Temperature Affecting Stereospecificity of Tandem DCRP ····· S7

VIII. SEC Traces of Polymers ····· S9

IX. Metal Sensing Experiments ····· S10

X. TGA and DCS Profiles of Polymers ····· S12

XI. ^{1}H and ^{13}C NMR Spectra of Monomers and Polymers ····· S13

XII. References ····· S18

I. General Methods

Materials

All reagents which are commercially available were used without further purification except for monomers. To avoid unwanted impurities in monomers, recrystallization and flash column chromatography were conducted before using each monomer. Solvents for starting materials synthesis were also commercially obtained. Thin-layer chromatography (TLC) was carried out on MERCK TLC silica gel 60 F254 and flash column chromatography was performed using MERCK silica gel 60 (0.040–0.063 mm). For metal sensing experiments, the solutions of the metal ions were prepared from their nitrate salts

Characterization

^{1}H NMR and *^{13}C NMR spectroscopies* were recorded by Varian/Oxford As-500 (125 MHz for ^{13}C NMR) and Agilent 400-MR (5400 MHz for ^{1}H NMR and 100 MHz for ^{13}C NMR) spectrometers. *High resolution mass spectroscopy (HRMS)* analyses were performed by the ultrahigh resolution ESI Q-TOF mass spectrometer (Bruker, Germany) in the Sogang Centre for Research Facilities. *Size exclusion chromatography (SEC)* analyses were carried out with Waters system (1515 pump and 2707 autosampler) and Shodex GPC LF-804 column eluted with HPLC-grade THF and filtered with a 0.2 μm PTFE filter (Whatman). The flow rate was 1.0 mL/min and temperature of column was maintained at 35 °C. For molecular weight characterization Wyatt DAWN-HELEOS 8+ multi-angle light scatter (MALS) and Wyatt OptiLab T-rEx refractive index (RI) detectors (both maintained at 35 ºC) were used. Molecular weights were determined from light scattering using *dn/dc* values calculated from batch mode measurements of polymer solutions at different concentrations. *Optical rotations* were obtained at 589 nm using a JASCO polarimeter. *Circular dichroism (CD) spectra* were taken with an AppliedPhotophysics Inc. circular dichroism detector Chirascan plus in National Instrumentation Center for Environmental Management (NICEM). *UV-vis spectra* were recorded on V-650 spectrometer with Jasco Inc. software. *Fluorescence spectra* were recorded on a Photon Technology International (PTI) QM-400 spectrofluorometer with FelixGX software. *Thermogravimetric analysis (TGA)* and differential scanning calorimetry (TGA) were carried out under N_2 gas at a scan rate of 10 °C/min with Q50 model device (TA Instruments).

II. Preparation and Characterization of Monomers

4,4'-(((2,5-bis((2-ethylhexyl)oxy)-1,4-phenylene)bis(methylene))bis(oxy))bis(2-methoxybenzaldehyde) (2b)

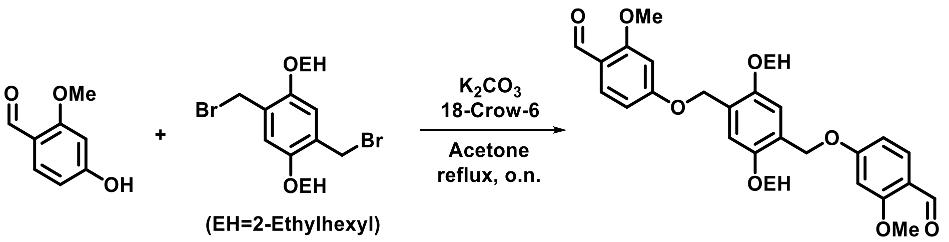

2b

2b was prepared by the slightly modified procedure from the previous literature.[1] To a solution of 4-hydroxy-2-methoxybenzaldehyde (1.94 g, 12.5 mmol) in acetone (16.7 mL) were added K_2CO_3 (2.08 g, 15 mmol), 18-crown-6 (0.27 g, 1.0 mmol). After stirring for 15 min at room temperature, 1,4-bis(bromomethyl)-2,5-bis((2-ethylhexyl)oxy)-benzene (2.6 g, 5.0 mmol)[2] was added to the reaction mixture. After being refluxed overnight, the mixture was cooled to room temperature, extracted with CH_2Cl_2 three times, dried over $MgSO_4$, and evaporated under reduced pressure to

afford crude product, which was purified by column chromatography on silica gel (*n*-hexane/EtOAc = 4/1, R_f = 0.3) to afford **2b** as white solid (2.89 g, 4.36 mmol, 87%); **^{1}H NMR** (400 MHz, $CDCl_3$) δ 10.29 (s, 2H), 7.80 (d, *J* = 8.7 Hz, 2H), 7.01 (s, 2H), 6.64 (dd, *J* = 8.7, 1.9 Hz, 2H), 6.54 (d, *J* = 2.0 Hz, 2H), 5.17 (s, 4H), 3.88 (s, 6H), 3.86 (s, 4H), 1.75–1.63 (m, 2H), 1.49–1.22 (m, 16H), 0.96–0.82 (m, 12H). **^{13}C NMR** (100 MHz, $CDCl_3$) δ 188.3, 165.5, 163.7, 150.6, 130.8, 125.2, 119.2, 112.7, 106.7, 98.8, 71.4, 65.4, 55.6, 39.6, 30.8, 29.2, 24.1, 23.1, 14.1, 11.3. **HRMS (ESI)** m/z: $[M + Na]^+$ Calcd for $C_{40}H_{54}O_8Na$ 685.3711; Found 685.3714.

4,4'-(hexane-1,6-diylbis(oxy))bis(2-methoxybenzaldehyde) (2c)

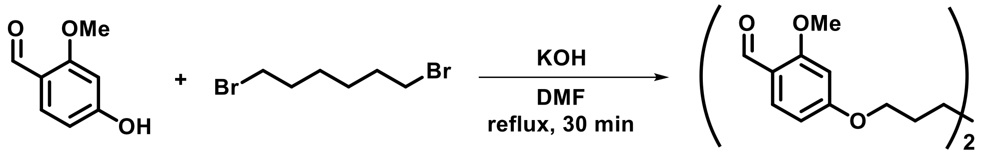

2c was prepared by the slightly modified procedure from the previous literature.[3] To a solution of 4-hydroxy-2-methoxybenzaldehyde (1.55 g, 10 mmol) in DMF (5.0 ml) was added potassium hydroxide (0.59 g, 10 mmol). After stirring for 30 min at room temperature, 1,6-dibromohexane (0.78 ml, 5.0 mmol) was added to the reaction mixture. The mixture was refluxed for 30 min to give **2c** as a white precipitate, then cooled to room temperature and poured onto ice/water. The solid was filtered and washed several times with water to afford crude product, which was further purified by recrystallization ($CHCl_3$/methanol) to afford **2c** as white solid (1.63 g, 4.22 mmol, 84%); **^{1}H NMR** (400 MHz, $CDCl_3$) δ 10.27 (s, 2H), 7.78 (d, *J* = 8.7 Hz, 2H), 6.52 (dd, *J* = 8.7, 1.9 Hz, 2H), 6.43 (d, *J* = 2.0 Hz, 2H), 4.04 (t, *J* = 6.4 Hz, 4H), 3.88 (s, 6H), 1.91–1.78 (m, 4H), 1.63–1.49 (m, 4H). **^{13}C NMR** (100 MHz, $CDCl_3$) δ 188.4, 165.8, 163.7, 130.9, 119.1, 106.2, 98.5, 68.3, 55.7, 29.2, 25.9. **HRMS (ESI)** m/z: $[M + Na]^+$ Calcd for $C_{22}H_{26}O_6Na$ 409.1622; Found 409.1624.

***meso*-1,2-bis(2-hydroxyphenyl)-ethylenediamine (*meso*-1)**

***meso*-1** was prepared according to the previously reported synthetic method.[4]

III. General Procedure for the Polymerization and Characterization of Polymers

To a 4 mL sized vial with a magnetic bar, 4 Å molecular sieve (0.3 g), diamine (**1**, 0.2 mmol), bis-benzaldehyde (**2**, 0.2 mmol, 1.0 equiv), *p*-toluenesulfonic acid (*p*-TsOH), and experimental solvent (0.8 mL, 0.25 M) were added. The reaction mixture was stirred at experimental temperature during the experimental reaction time, then precipitated in isopropyl alcohol (IPA) at room temperature. The obtained solid was filtered and dried in vacuo.

(*R*,*R*)-P1 (synthesized from **(*S*,*S*)-1** at rt)

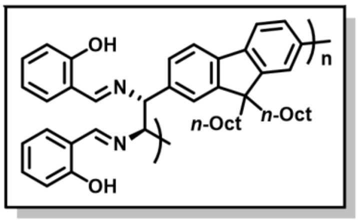

^{1}H NMR (400 MHz, CD_2Cl_2) δ 13.21 (s, 2H), 8.28 (s, 2H), 7.58–6.41 (m, 14H), 4.81 (s, 2H), 1.78 (br, 4H), 1.31–0.63 (m, 26H), 0.39 (br, 4H). **^{13}C NMR** (100 MHz, $CDCl_3$) δ 166.1, 161.1, 151.4, 140.2, 138.6, 132.6, 131.7, 127.0, 122.1, 119.6, 118.8, 118.7, 117.0, 80.7, 55.4, 55.1, 40.2, 31.9, 30.1, 29.5, 24.0, 22.8, 14.3. $[\alpha]_D^{24}$ +135.95 (c = 1.0 in $CHCl_3$).

(*S*,*S*)-P1 (synthesized from **(*R*,*R*)-1** at rt)

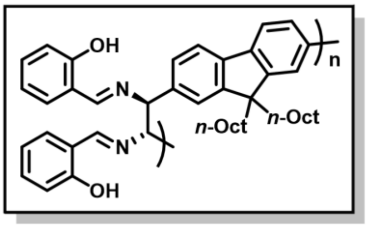

^{1}H NMR (400 MHz, CD_2Cl_2) δ 13.20 (s, 2H), 8.28 (s, 2H), 7.58–6.41 (m, 14H), 4.80 (s, 2H), 1.78 (br, 4H), 1.31–0.63 (m, 26H), 0.39 (br, 4H). **^{13}C NMR** (100 MHz, $CDCl_3$) δ. 166.1, 161.1, 151.4, 140.2, 138.6, 132.6, 131.7, 127.0, 122.1, 119.6, 118.8, 118.7, 117.0, 80.7, 55.4, 55.1, 40.2, 31.9, 30.1, 29.5, 24.0, 22.8, 14.3. **$[\alpha]_D^{24}$** -134.71 (c = 1.0 in $CHCl_3$).

(*R*,*R*)-*r*-(*S*,*S*)-P1 (synthesized from racemic mixture of **1** at rt)

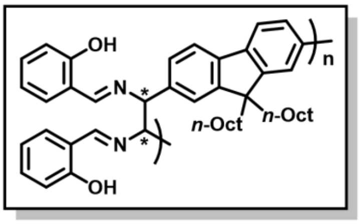

^{1}H NMR (400 MHz, CD_2Cl_2) δ 13.21 (s, 2H), 8.27 (s, 2H), 7.58–6.41 (m, 14H), 4.80 (s, 2H), 1.78 (br, 4H), 1.31–0.14 (m, 30H). **^{13}C NMR** (100 MHz, $CDCl_3$) δ 166.1, 161.1, 151.4, 140.2, 138.6, 132.6, 131.7, 127.0, 122.2, 119.6, 118.8, 118.7, 117.0, 80.7, 55.4, 55.0, 40.2, 31.9, 30.1, 29.5, 24.1, 22.8, 14.3. **$[\alpha]_D^{24}$** -0.27 (c = 1.0 in $CHCl_3$).

(*R*,*R*)-P2

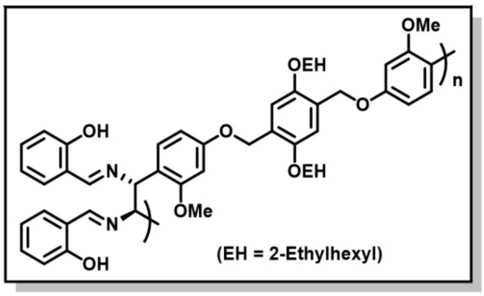

^{1}H NMR (400 MHz, $CDCl_3$) δ 13.78 (s, 2H), 8.21 (s, 2H), 7.24–6.23 (m, 16H), 5.28 (s, 2H), 4.99 (s, 4H), 3.96–3.58 (m, 10H), 1.64 (br, 2H), 1.49–1.16 (m, 16H), 0.84 (t, 12H). **^{13}C NMR** (125 MHz, $CDCl_3$) δ 165.7, 161.5, 159.4, 157.6, 150.5, 132.2, 131.6, 130.0, 125.7, 121.0, 119.0, 118.4, 117.1, 112.5, 105.4, 99.2, 71.4, 71.2, 65.2, 55.5, 39.7, 30.8, 29.2, 24.2, 23.1, 14.2, 11.3. **$[\alpha]_D^{24}$** +10.23 (c = 1.0 in $CHCl_3$).

(*R*,*R*)-P3

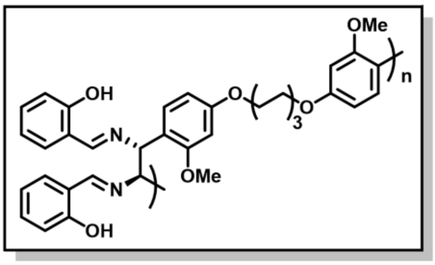

^{1}H NMR (400 MHz, $CDCl_3$) δ 13.79 (s, 2H), 8.20 (s, 2H), 7.24–6.12 (m, 14H), 5.27 (s, 2H), 4.10–3.52 (m, 10H), 1.72 (br, 4H), 1.45 (br, 4H). **^{13}C NMR** (125 MHz, $CDCl_3$) δ 165.7, 161.6, 159.5, 157.7, 132.2, 131.6, 129.9, 120.8, 119.0, 118.4, 117.1, 105.0, 99.0, 71.1, 68.0, 55.5, 29.4, 26.0. **$[\alpha]_D^{24}$** +8.56 (c = 1.0 in $CHCl_3$).

P4

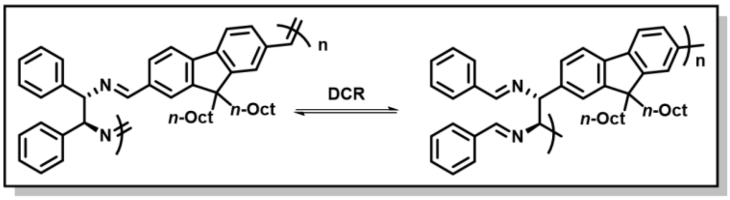

^{1}H NMR (400 MHz, CD_2Cl_2) δ 8.29 (s, 2H), 8.00–6.67 (m, 16H), 4.77 (s, 2H), 1.91 (br, 4H), 1.40–0.12 (m, 30H). **^{13}C NMR** could not be obtained due to hydrolysis during the measurement. **$[\alpha]_D^{24}$** +172.91 (c = 1.0 in $CHCl_3$).

IV. Optimization of Polymerization

Table S1. Optimization of Catalyst Loading for (*R*,*R*)-P1

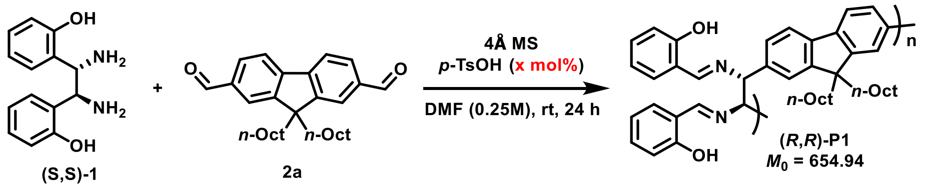

Entry	*p*-TsOH (mol%)	Conv (%)[a]	M_n ($Đ$)[b]	Yield (%)[c]
1	10	96	14.9k (1.40)	64
2	15	96	16.6 k (1.88)	99
3	20	97	22.3 k (1.87)	80
4	25	95	14.2 k (1.69)	78
5	50	95	15.5 k (1.69)	90
6	100	96	16.9k (1.60)	99

Table S2. Optimization of Catalyst Loading for (*R*,*R*)-P2

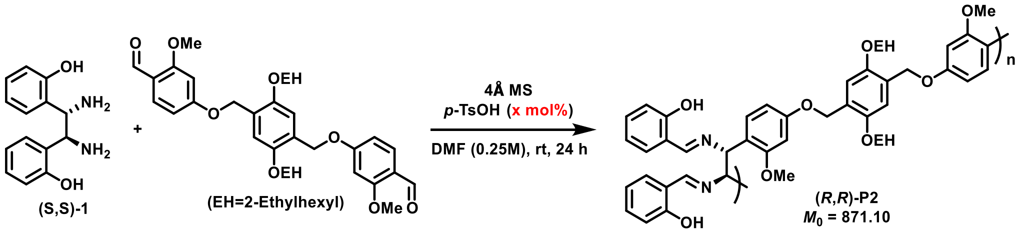

Entry	*p*-TsOH (mol%)	Conv (%)[a]	M_n ($Đ$)[b]	Yield (%)[c]
1	20	97	33.3 k (1.61)	90
2	25	96	19.9 k (1.74)	85
3	30	95	18.5 k (1.94)	98

Table S3. Optimization of Reaction Temperature for (*R*,*R*)-P3

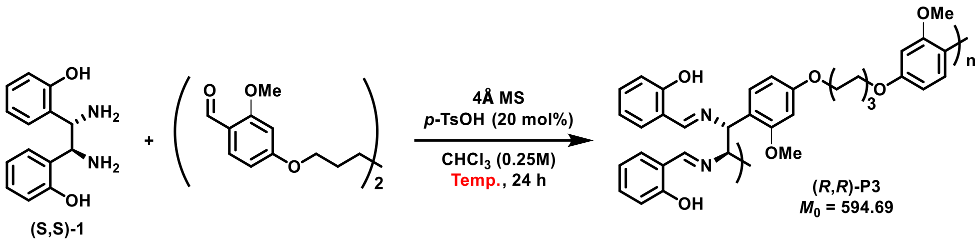

Entry	Temp. (°C)	Conv (%)[a]	M_n ($Đ$)[b]	Yield (%)[c]
1	rt	96	14.6 k (2.45)	79
2	40	97	16.7 k (1.76)	78
3	60	95	10.0 k (1.66)	77

[a]Determined by ^{1}H NMR analysis of the crude reaction mixture. [b]Absolute molecular weights determined by THF SEC using a multiangle laser light scattering (MALLS) detector. [c]Isolated yields after purification from IPA.

V. *In situ* ^{1}H NMR Experiment in DMF-d_7

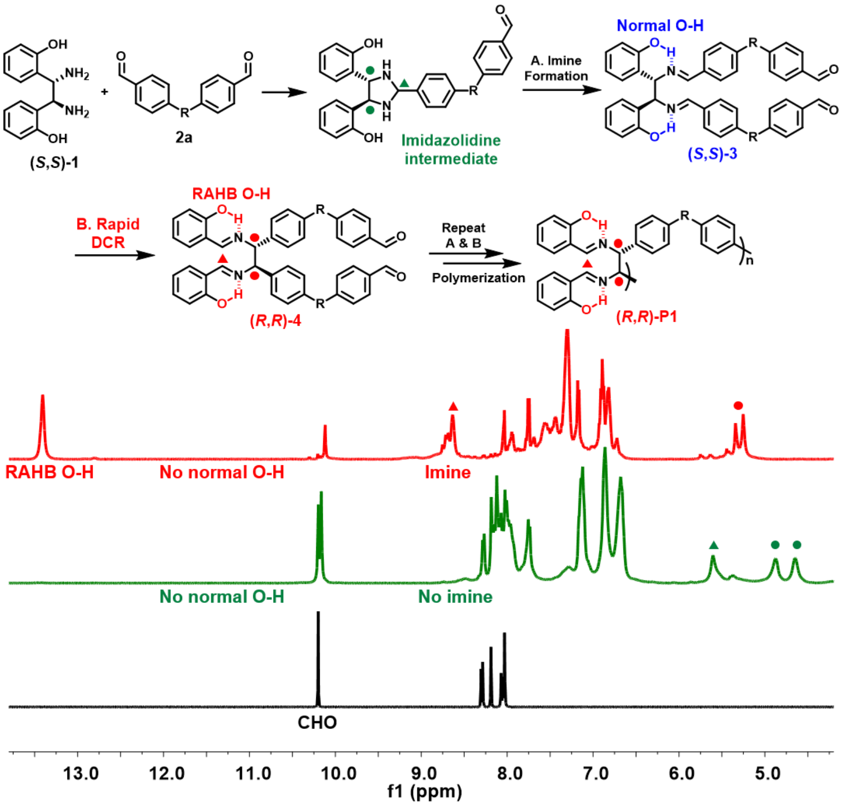

Figure S1. *In situ* ^{1}H NMR monitoring for the formation of imidazole intermediate before DCR in early stage of the polymerization in DMF-d_7. Assignment of peaks corresponding imidazolidine intermediate is supported by the previous literature.[5]

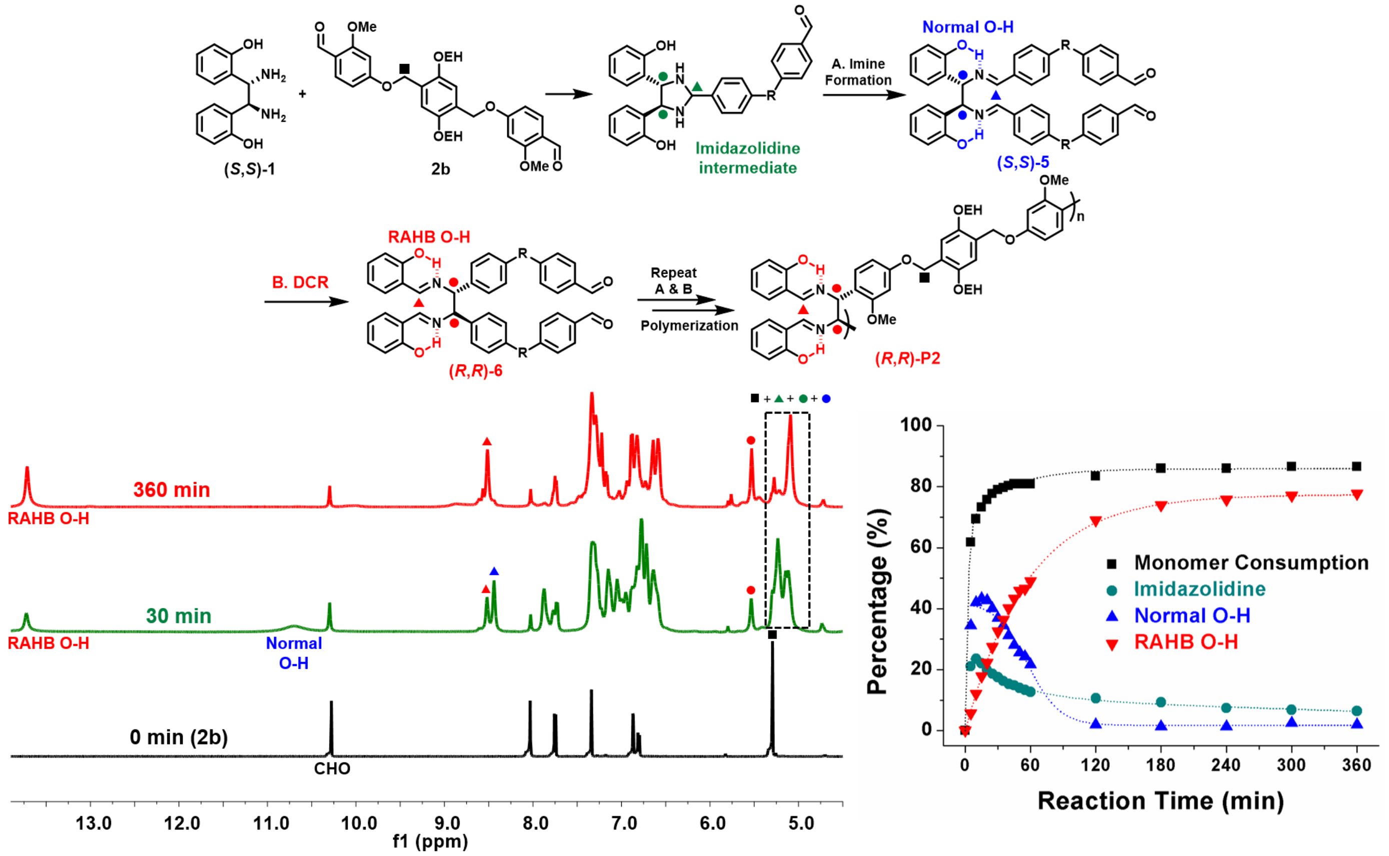

Figure S2. Monitoring the polymerization using **(*S,S*)-1** and **2b** by *in situ* ^{1}H NMR spectroscopy in DMF-d_7

VI. Control Experiment to Compare Stability against Hydrolysis

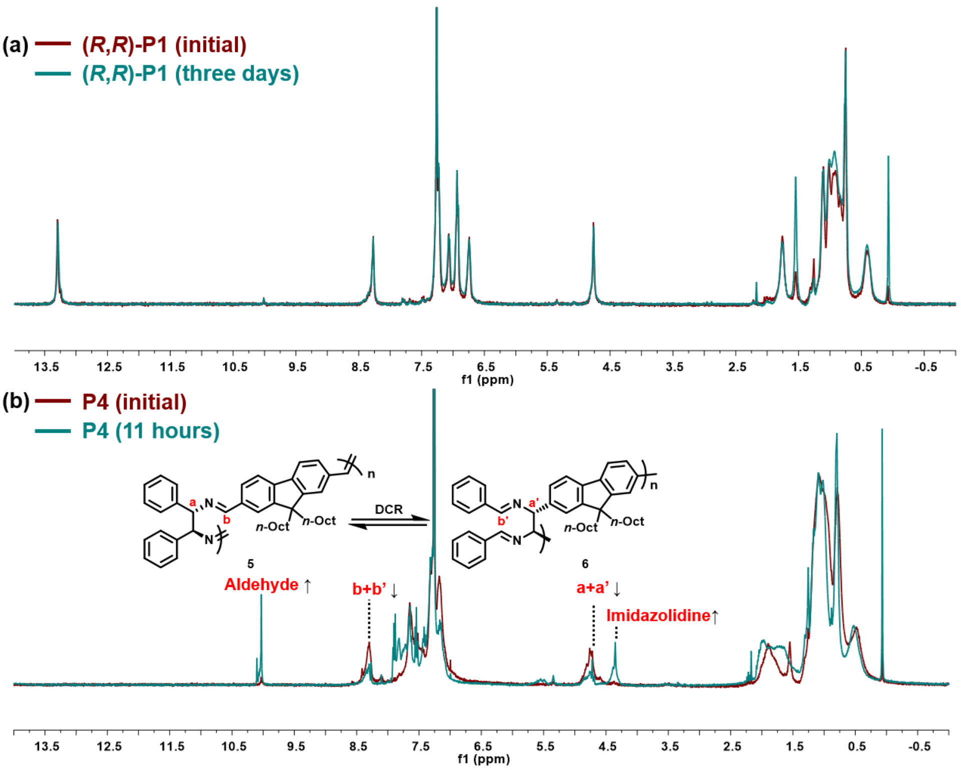

Figure S3. (a) ^{1}H NMR spectra of ***(R,R)*-P1** measured at the initial and three days after dissolving in $CDCl_3$. (b) ^{1}H NMR spectra of **P4** measured at the initial and 11 hours after dissolving in $CDCl_3$.

VII. Temperature Affecting Stereospecificity of Tandem DCRP

Table S4. Various P1 Synthesis by Varying Reaction Temperature and Configuration of 1

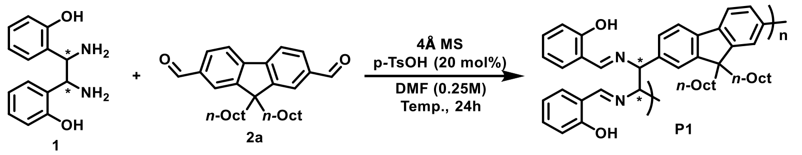

Entry	Configuration of 1	Temp. (°C)	$[\alpha]_D^{24}$ [a]	Conv (%)[b]	M_n ($Đ$)[c]	Yield (%)[d]
1	(*S*,*S*)	10	+134.47	95	13.9 k (1.60)	70
2	(*S*,*S*)	rt	+135.95	97	22.3 k (1.87)	80
3	(*S*,*S*)	50	+82.82	97	24.0 k (2.85)	99
4	(*S*,*S*)	70	+71.92	98	30.6 k (4.13)	99
5	(*S*,*S*)	90	+57.11	98	42.9 k (5.06)	99
6[e]	(*S*,*S*)	120	+53.68	94	35.8 k (2.20)	65
7[e]	(*S*,*S*)	140	+25.92	91	23.4 k (1.82)	62
8	(*R*,*R*)	rt	-134.71	97	20.1 k (2.34)	98
9	(*S*,*S*) + (*R*,*R*) (1:1)	rt	-0.27	97	21.1 k (2.77)	98
10	*meso*	rt	0	63	6.9 k (1.17)	65
11	*meso*	50	0	83	9.4 k (1.60)	71

[a]c = 1.0 in $CHCl_3$. [b]Determined by ^{1}H NMR analysis of the crude reaction mixture. [c]Absolute molecular weights determined by THF SEC using a multiangle laser light scattering (MALLS) detector. [d]Isolated yields after purification from IPA. [e]Polymerization proceeded for 5 h.

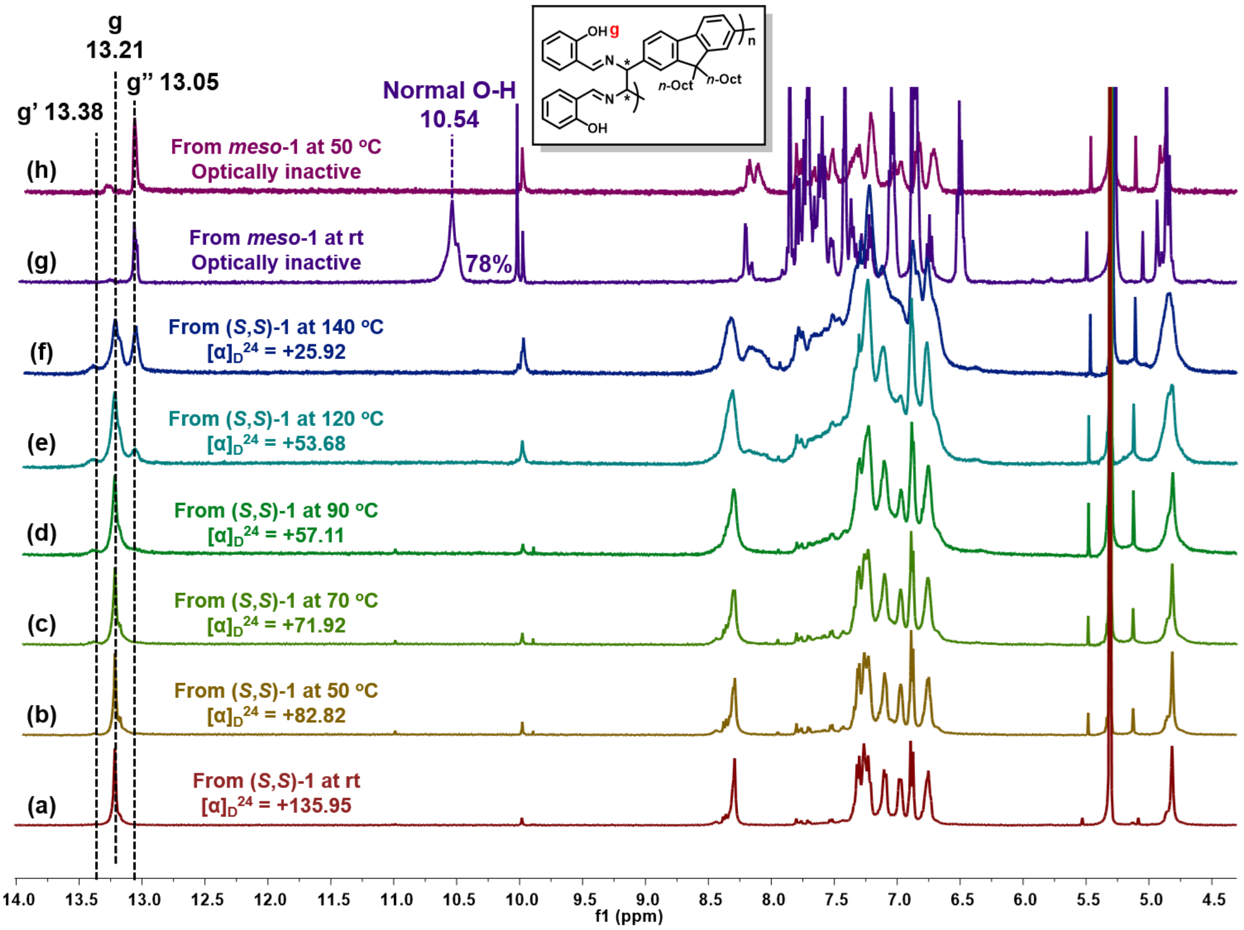

Figure S4. ^{1}H NMR spectra (in CD_2Cl_2) of various **P1** (in Table S4) synthesized from chiral and *meso* diamines at various reaction temperatures.

(a)

Configuration of **1**	Reaction Temp. (°C)	g (cc)	g' (cm)	g'' (mm)	Content of chiral salen unit (%)	$[\alpha]_D^{24}$
(*S*,*S*)	rt	100 %	0 %	0 %	100	+135.95
(*S*,*S*)	50	91.0 %	4.5 %	4.5 %	93.25	+82.82
(*S*,*S*)	70	86.9 %	6.1 %	7.0 %	87.95	+71.92
(*S*,*S*)	90	83.3 %	2.5 %	14.2 %	84.55	+57.11
(*S*,*S*)	120	72.5 %	12.3 %	15.2 %	78.65	+43.68
(*S*,*S*)	140	56.2 %	9.0 %	34.8 %	60.7	+25.92
meso	rt	0 %	0 %	100 %	0	0

(b)

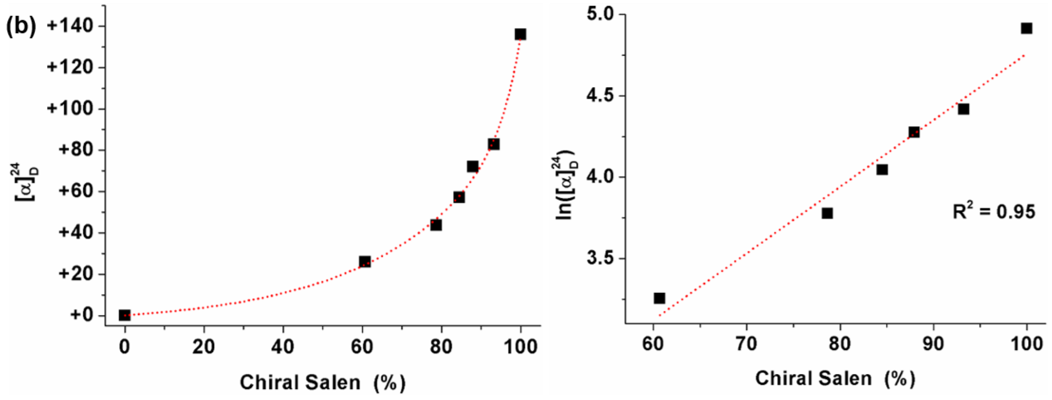

Figure S5. (a) Table for integrations of all RAHB O–H signals (g, g', and g'' peaks) and optical rotation values depending on reaction temperature. (b) Plots showing relationship between $[\alpha]_D^{24}$ and content of chiral salen unit determined by ^{1}H NMR spectra (in CD_2Cl_2). Chiral salen (%) was calculated by (g + 0.5*g')/(g + g' + g'').

VIII. SEC Traces of Polymers

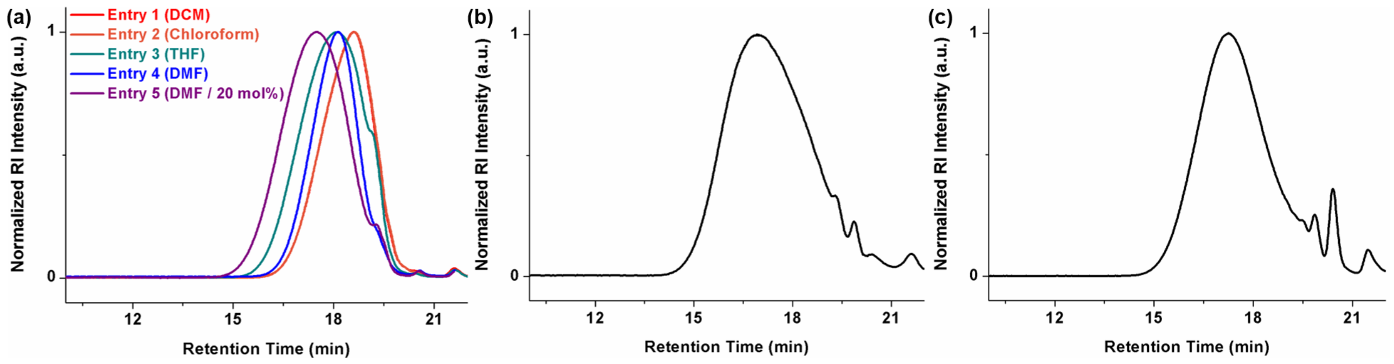

Figure S6. SEC traces for polymers in **Table 1**. (a) **(*R*,*R*)-P1** (entries 1–5). (b) **(*R*,*R*)-P2** (entry 6). (c) **(*R*,*R*)-P3** (entry 7).

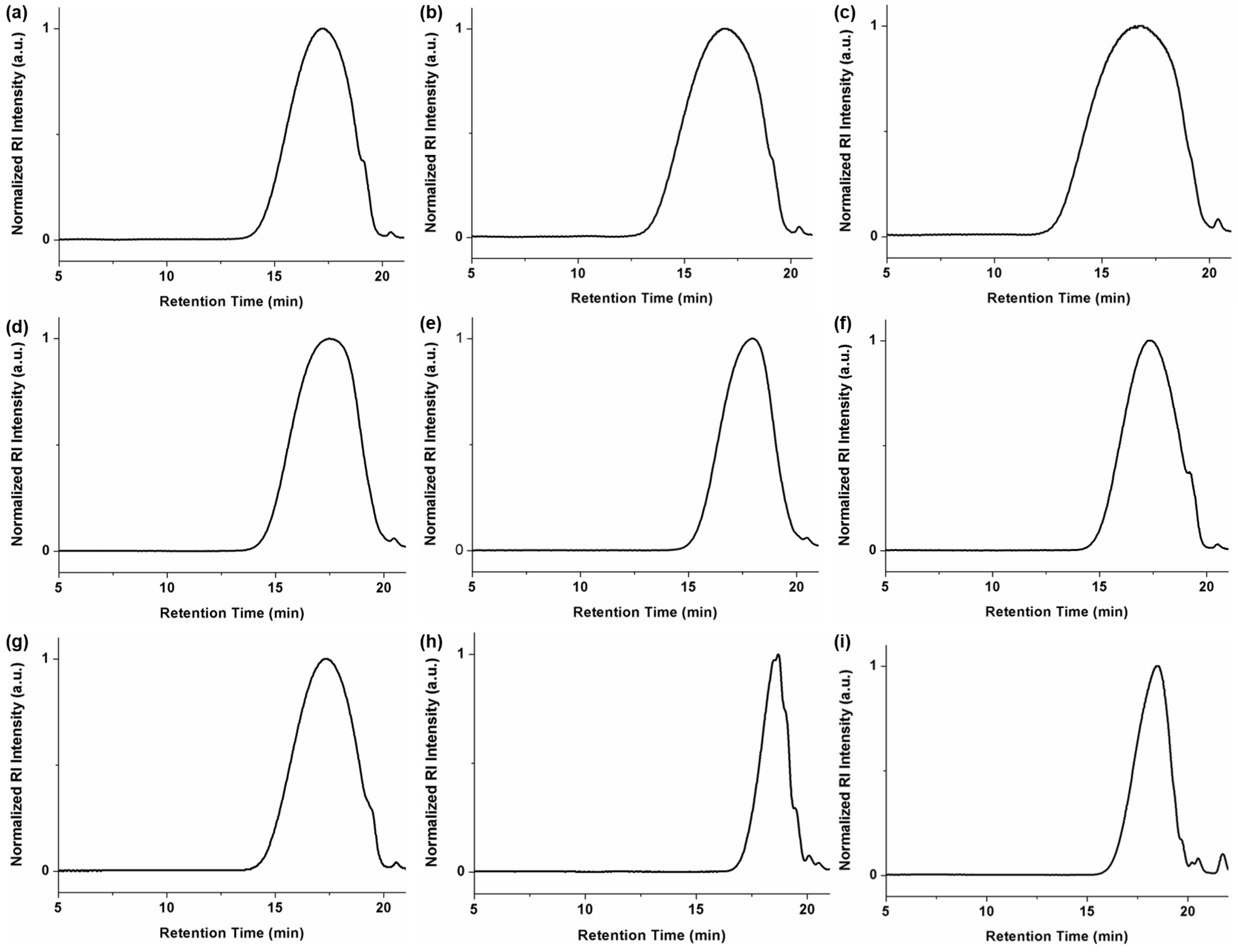

Figure S7. SEC traces for other polymers in **Table S4**. (a) **P1** synthesized at 50 °C from **(*S*,*S*)-1** (entry 2). (b) **P1** synthesized at 70 °C from **(*S*,*S*)-1** (entry 3). (c) **P1** synthesized at 90 °C from **(*S*,*S*)-1** (entry 4). (d) **P1** synthesized at 120 °C from **(*S*,*S*)-1** (entry 5). (e) **P1** synthesized at 140 °C from **(*S*,*S*)-1** (entry 6). (f) **P1** synthesized at room temperature from **(*R*,*R*)-1** (entry 7). (g) **P1** synthesized at room temperature from 1:1 mixture of **(*S*,*S*)-1** and **(*R*,*R*)-1** (entry 8). (h) **P1** synthesized at rt from ***meso*-1** (entry 9). (i) **P1** synthesized at 50 °C from ***meso*-1** (entry 10).

IX. Metal Sensing Experiments

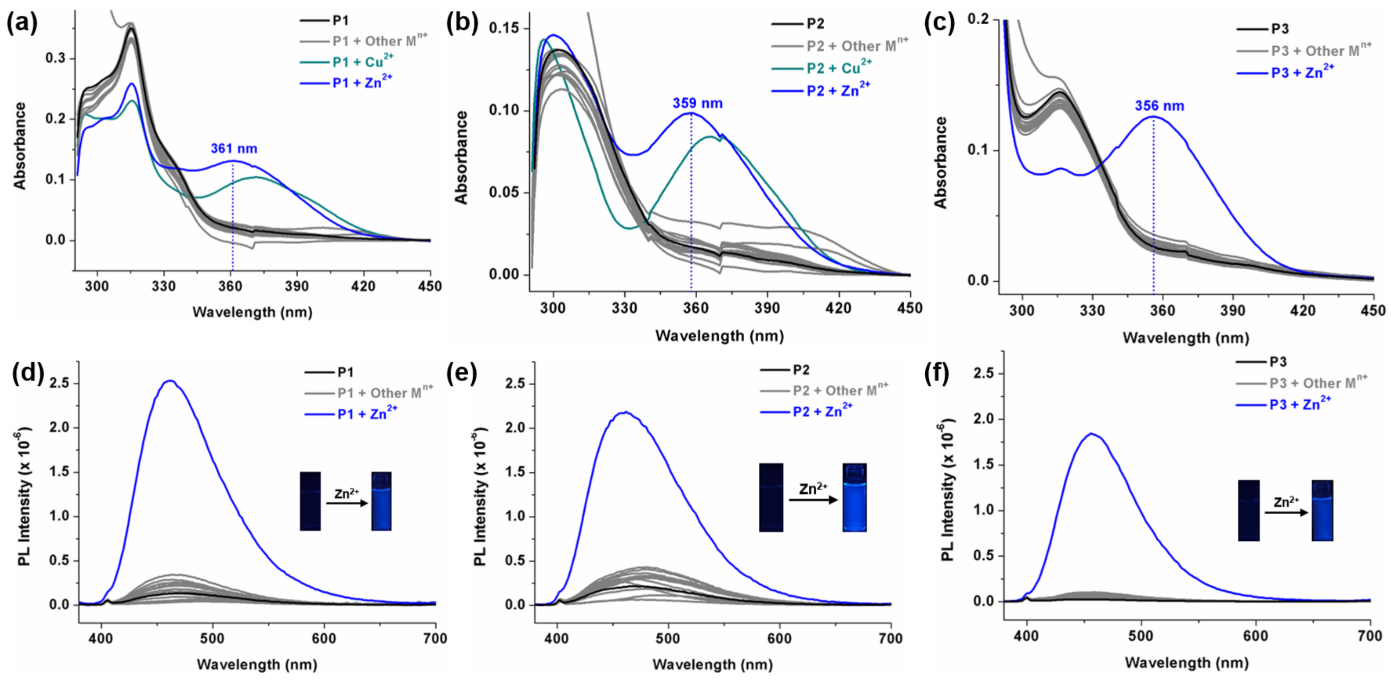

Figure S8. Absorption spectra of polymers: (a) **(*R*,*R*)-P1**, (b) **(*R*,*R*)-P2,** and (c) **(*R*,*R*)-P3**. Emission spectra of polymers: (d) **(*R*,*R*)-P1**, (e) **(*R*,*R*)-P2,** and (f) **(*R*,*R*)-P3** in THF:H_2O = 9:1 (v/v) solution (10 μM) at 298 K in the absence or presence of 1.0 equiv. of various metal cations.

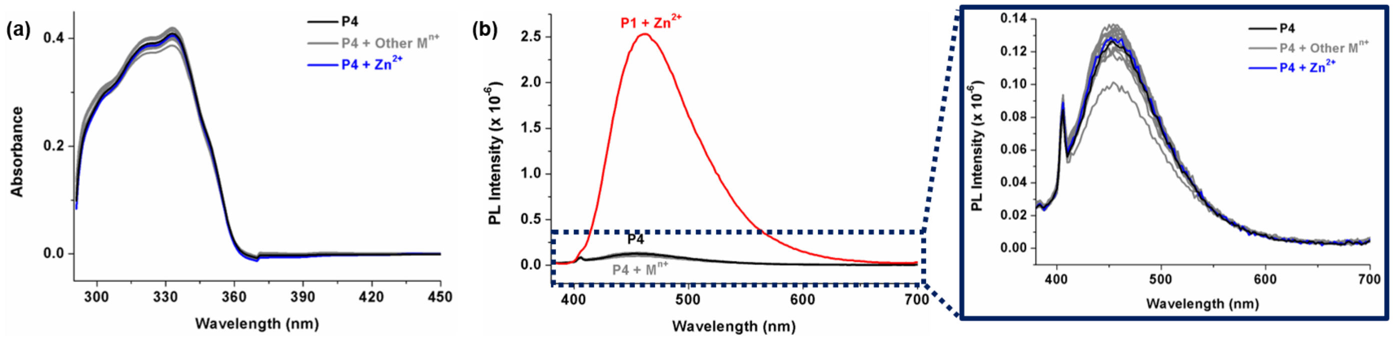

Figure S9. Control experiment to compare sensing ability. (a) Absorption and (b) emission spectra of **P4** in THF:H_2O = 9:1 (v/v) solution (10 μM) at 298 K in the absence or presence of 1.0 equiv. of various metal cations.

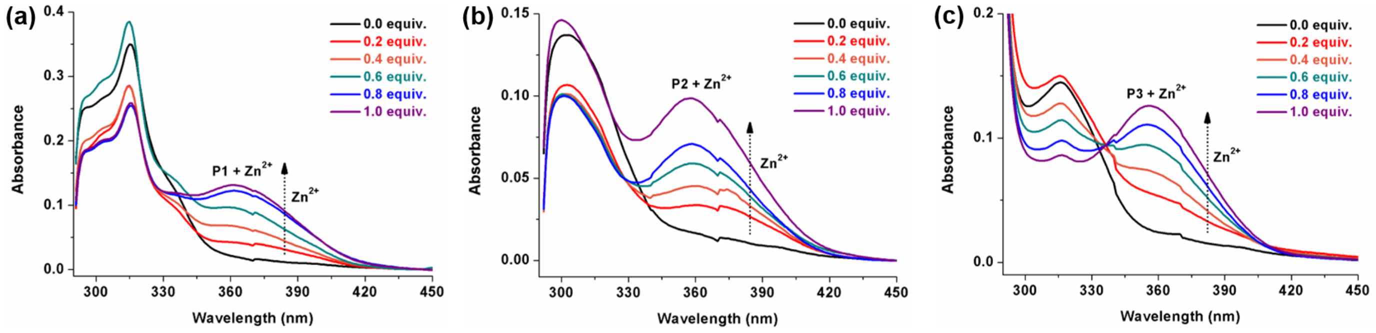

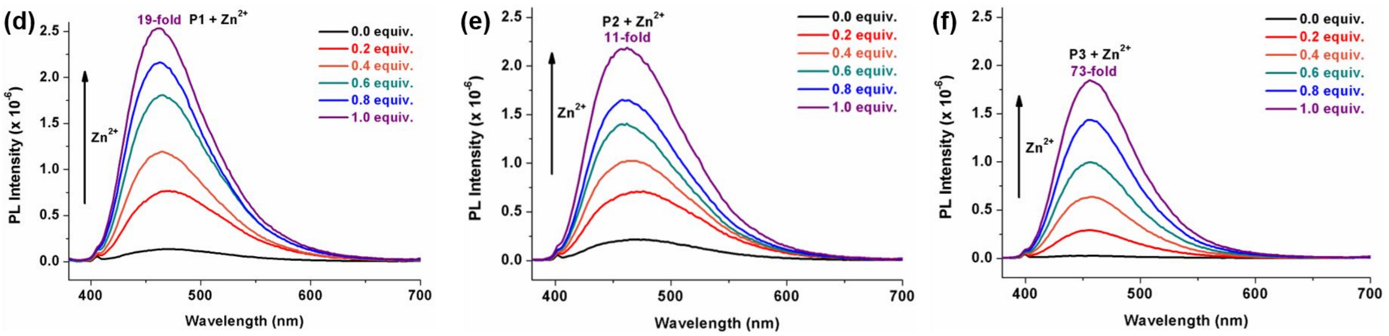

Figure S10. Absorption spectra of polymers: (a) **(*R*,*R*)-P1**, (b) **(*R*,*R*)-P2,** and (c) **(*R*,*R*)-P3**. Emission spectra of polymers: (d) **(*R*,*R*)-P1** ($I/I_0 = 19$), (e) **(*R*,*R*)-P2** ($I/I_0 = 11$), and (f) **(*R*,*R*)-P3** ($I/I_0 = 73$) in THF:H_2O = 9:1 (v/v) solution (10 μM) at 298 K with increasing amounts of Zn^{2+} (0.0–1.0 equiv.).

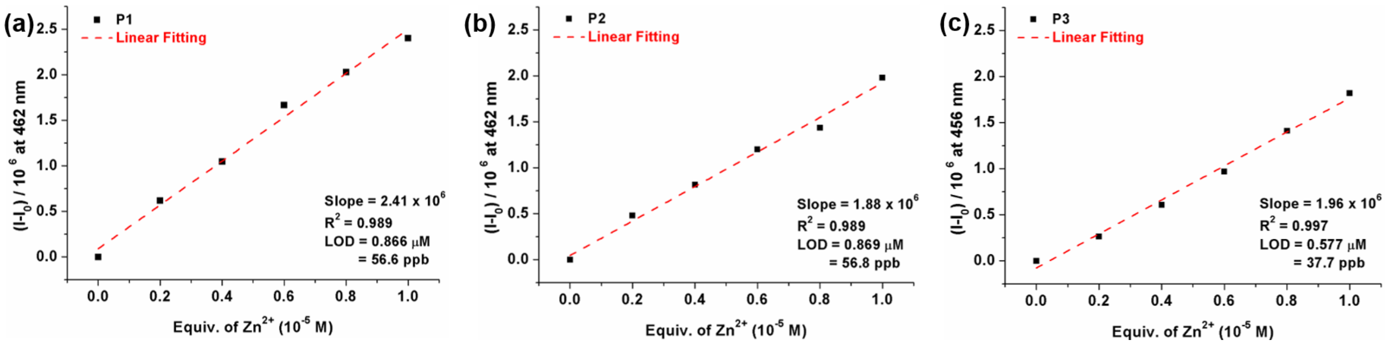

Figure S11. The linear relationship between $I-I_0$ and [Zn^{2+}]: (a) **(*R*,*R*)-P1**, (b) **(*R*,*R*)-P2**, and (c) **(*R*,*R*)-P3**.

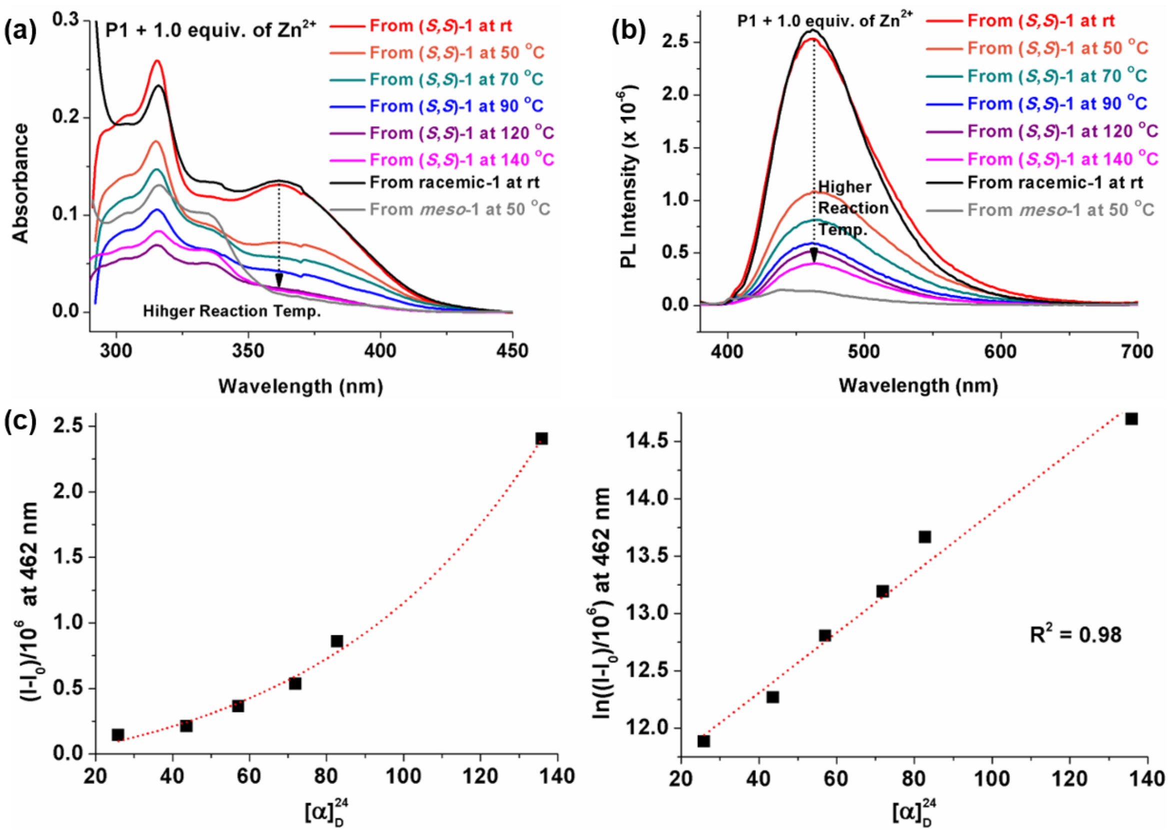

Figure S12. (a) Absorption and (b) emission spectra of various **P1** in THF:H_2O = 9:1 (v/v) solution (10 μM) at 298 K in the presence of 1.0 equiv. of Zn^{2+}. (c) Plots showing relationship between $I-I_0$ and $[\alpha]_D^{24}$.

X. TGA and DSC Profiles of Polymers

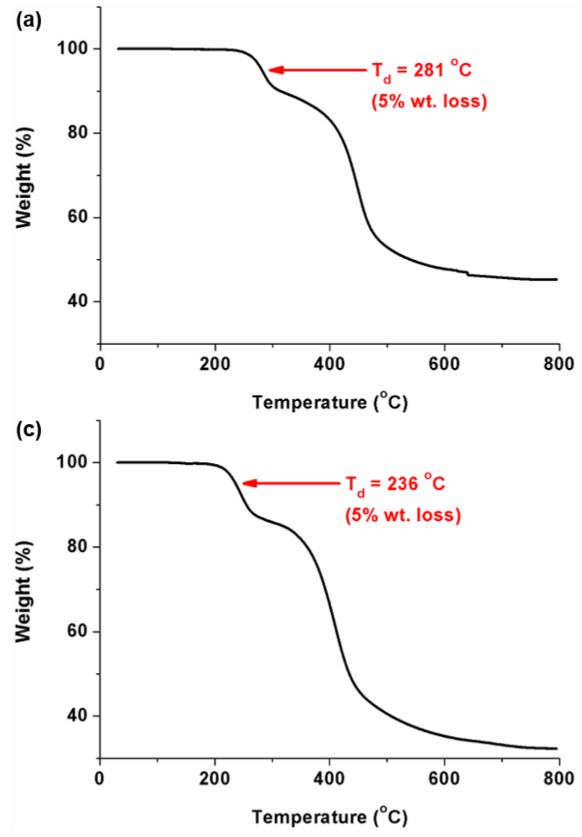

Figure S13. TGA profiles of (a) **(*R*,*R*)-P1**, (b) **(*R*,*R*)-P2**, (c) **(*R*,*R*)-P3**, and (d) **P4**.

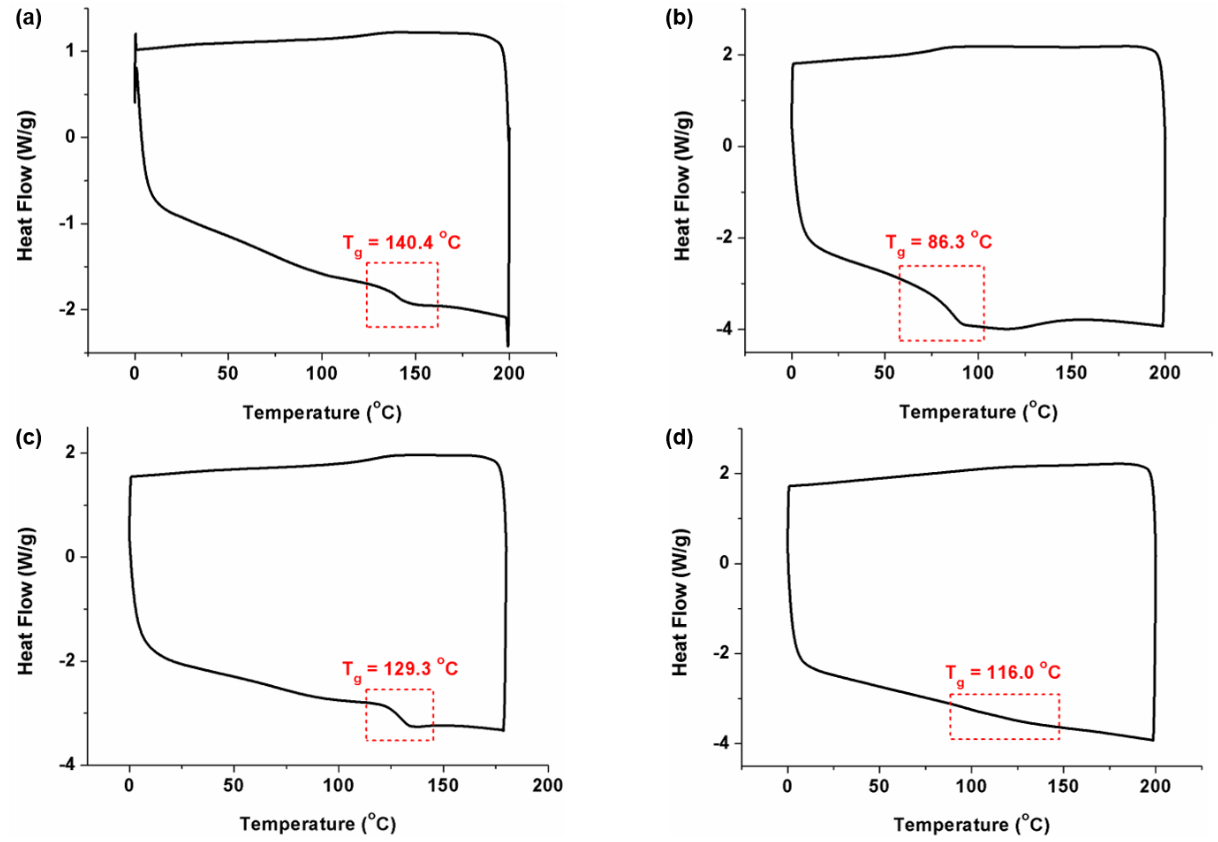

Figure S14. DSC profiles of (a) **(*R*,*R*)-P1**, (b) **(*R*,*R*)-P2**, (c) **(*R*,*R*)-P3**, and (d) **P4**.

XI. ^{1}H and ^{13}C NMR Spectra of Monomers and Polymers

2b ^{1}H NMR (400 MHz, $CDCl_3$)

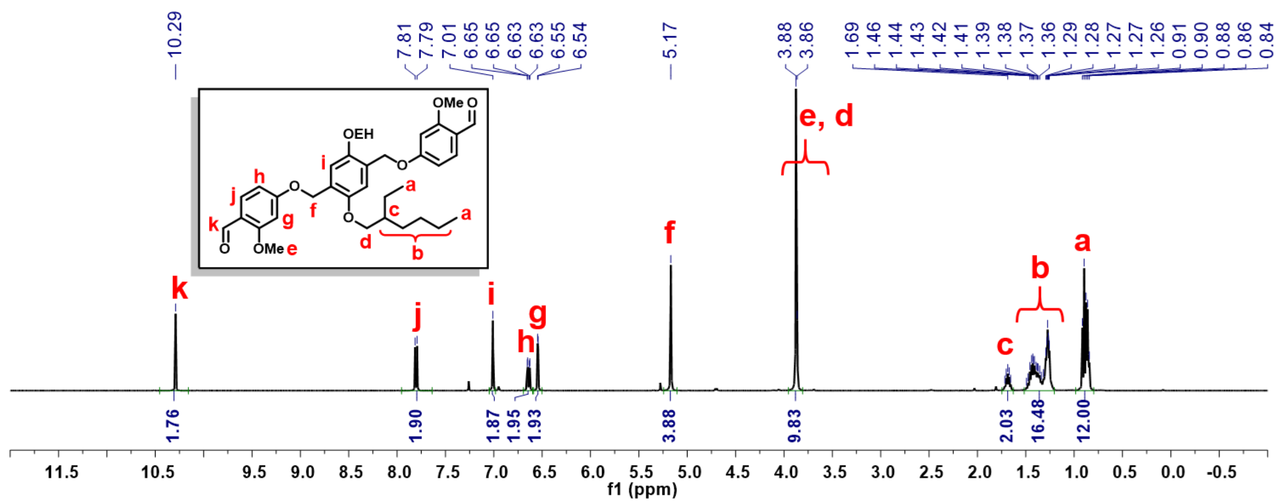

2b ^{13}C NMR (100 MHz, $CDCl_3$)

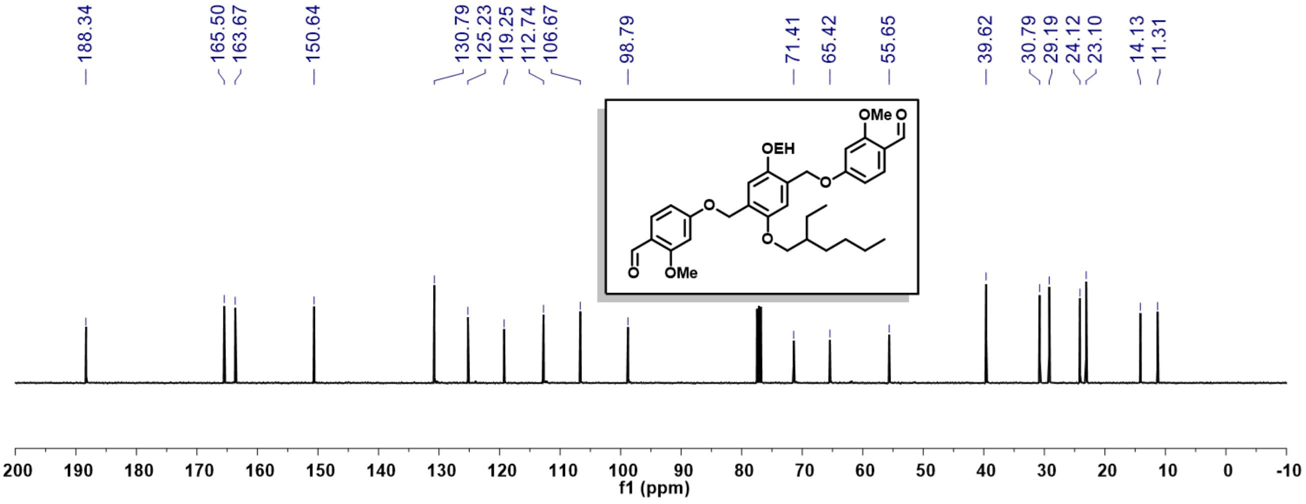

2c ^{1}H NMR (400 MHz, $CDCl_3$)

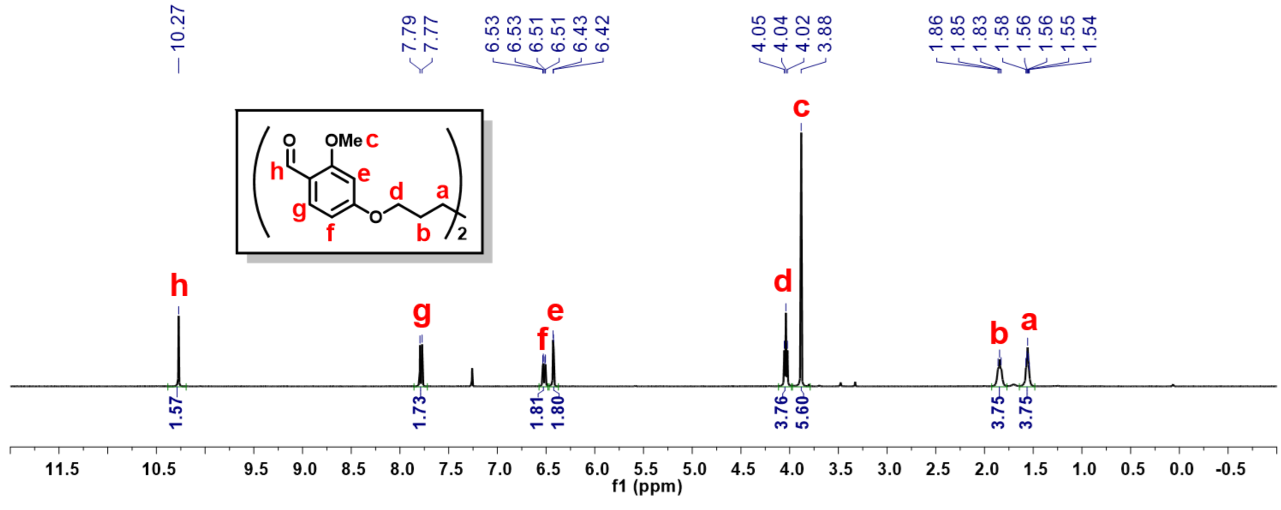

2c ^{13}C NMR (100 MHz, $CDCl_3$)

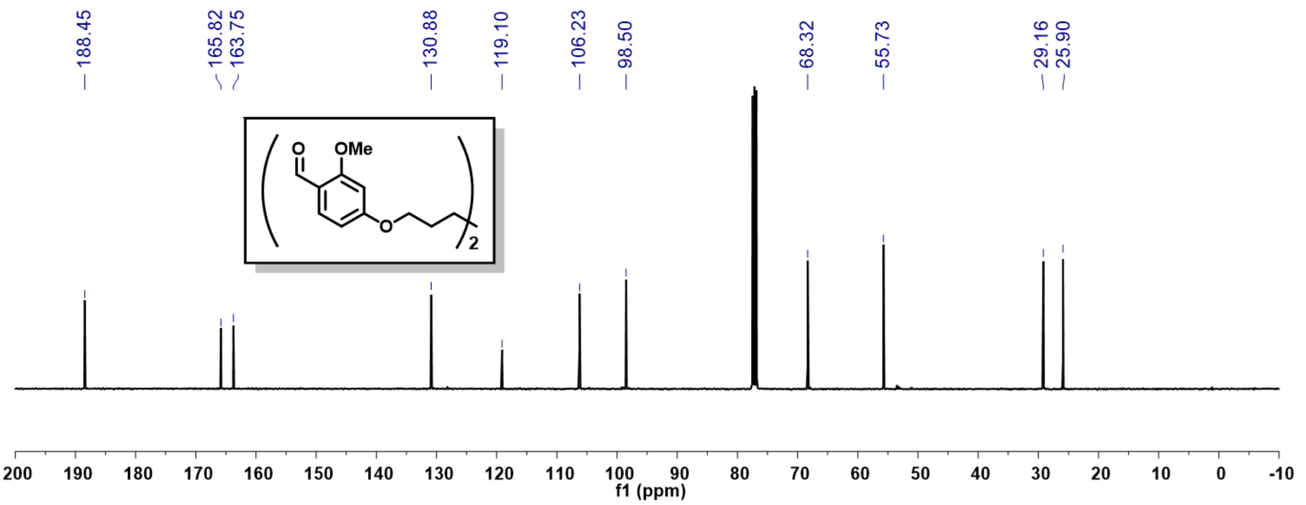

(***R***,***R***)**-P1** (synthesized from (***S***,***S***)**-1** at rt) ^{1}H NMR (400 MHz, $CDCl_3$)

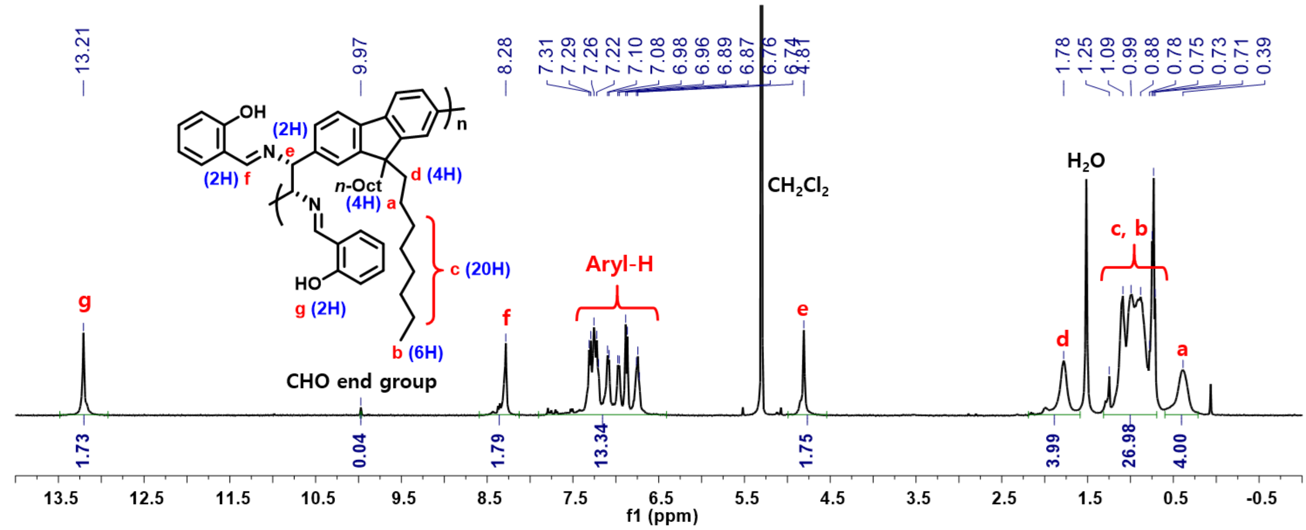

(***R***,***R***)**-P1** (synthesized from (***S***,***S***)**-1** at rt) ^{13}C NMR (100 MHz, $CDCl_3$)

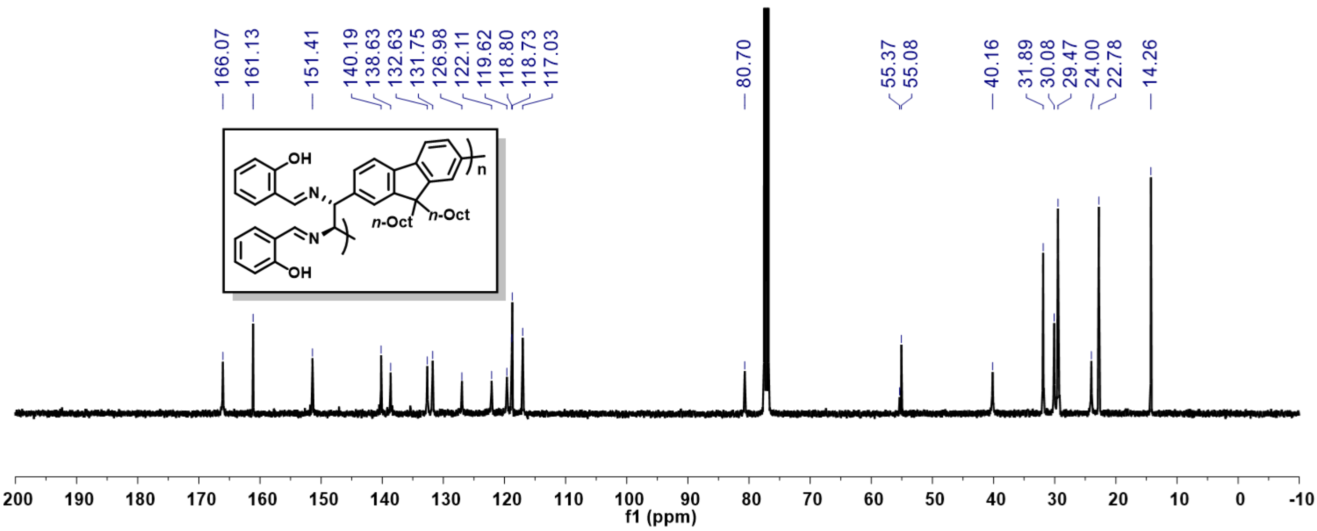

(***S*,*S***)**-P1** (synthesized from (***R*,*R***)**-1** at rt) ^{1}H NMR (400 MHz, $CDCl_3$)

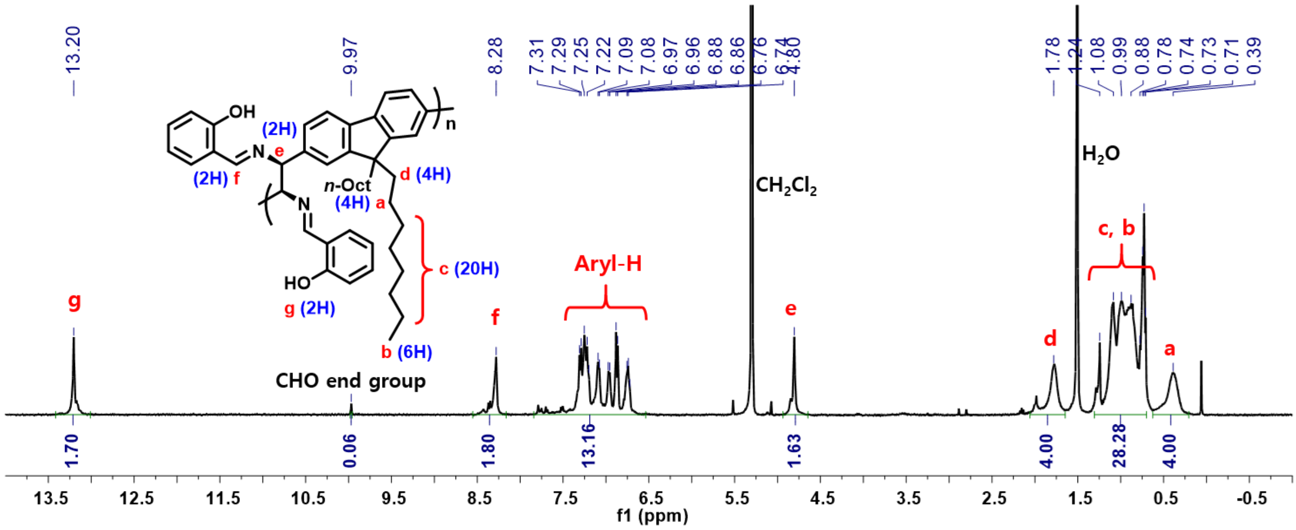

(***S*,*S***)**-P1** (synthesized from (***R*,*R***)**-1** at rt) ^{13}C NMR (100 MHz, $CDCl_3$)

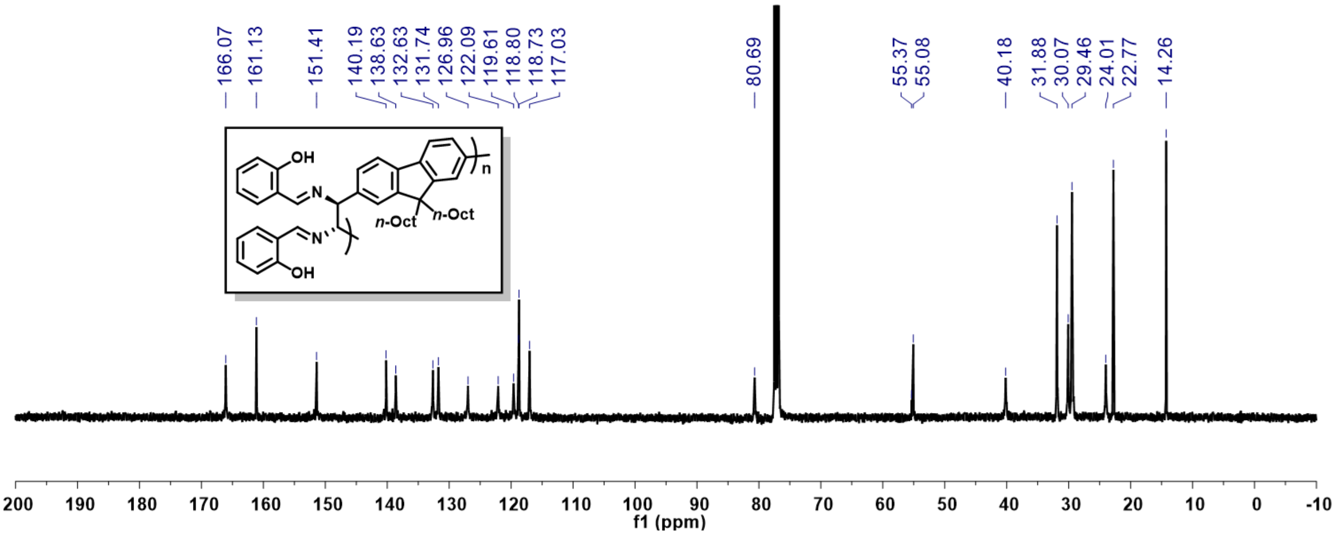

(***R*,*R***)**-*r*-(*S*,*S*)-P1** (synthesized from racemic mixture of **1** at rt) ^{1}H NMR (400 MHz, $CDCl_3$)

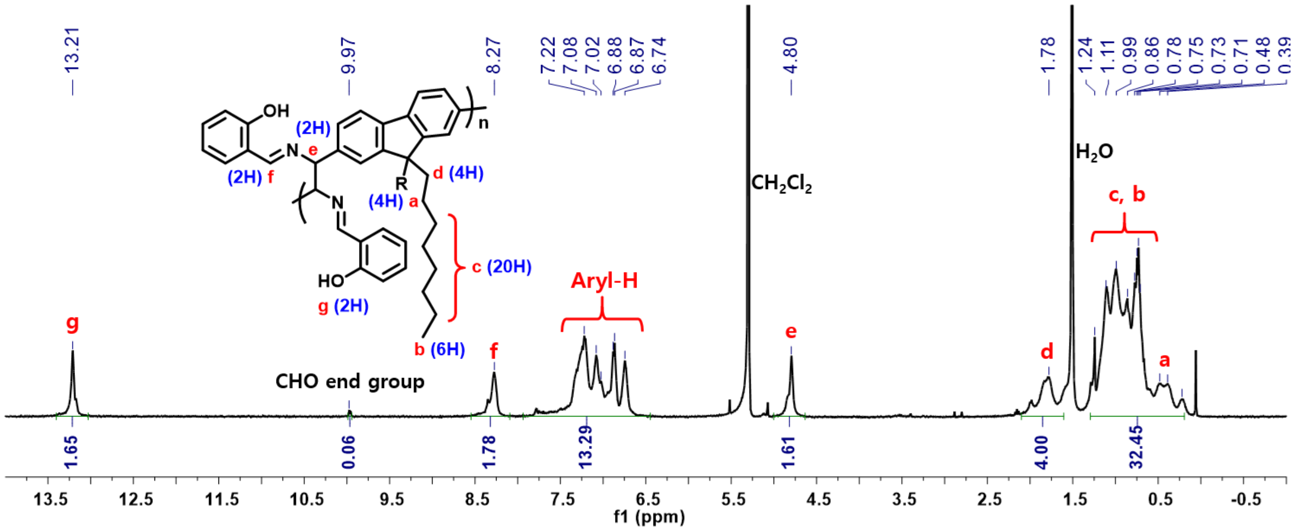

(***R***,***R***)-***r***-(***S***,***S***)-**P1** (synthesized from racemic mixture of **1** at rt) ^{13}C NMR (100 MHz, $CDCl_3$)

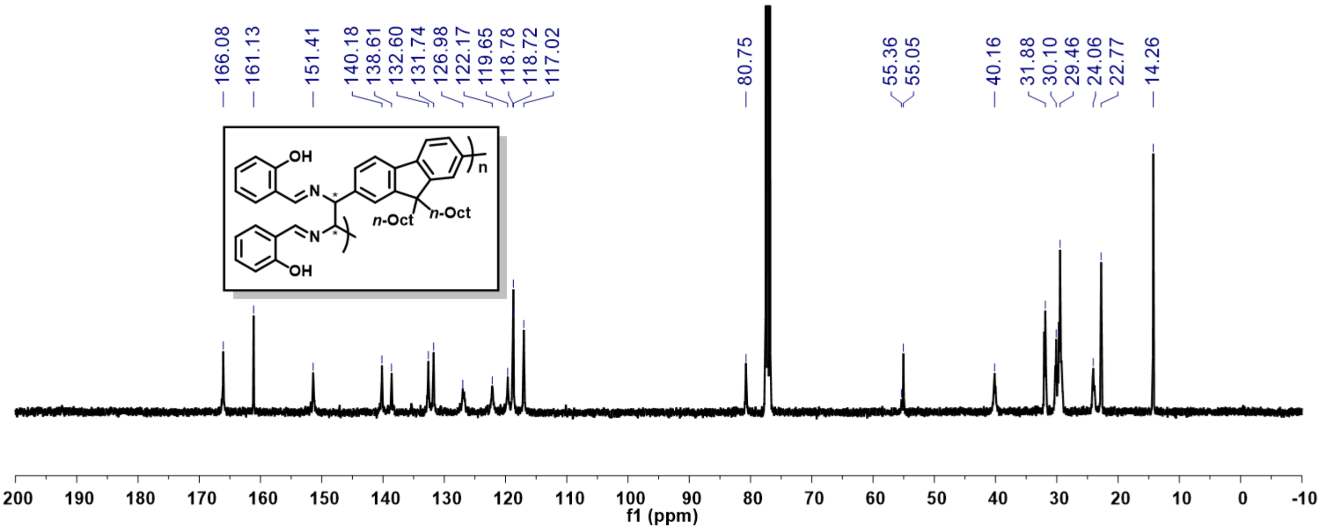

(***R***,***R***)-**P2** ^{1}H NMR (400 MHz, $CDCl_3$)

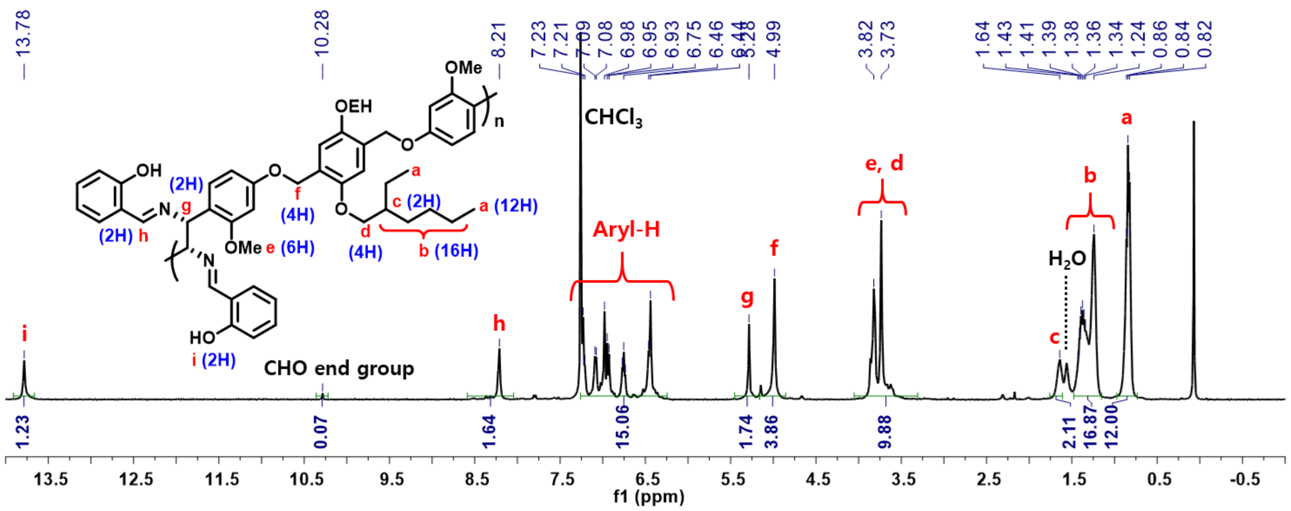

(***R***,***R***)-**P2** ^{13}C NMR (125 MHz, $CDCl_3$)

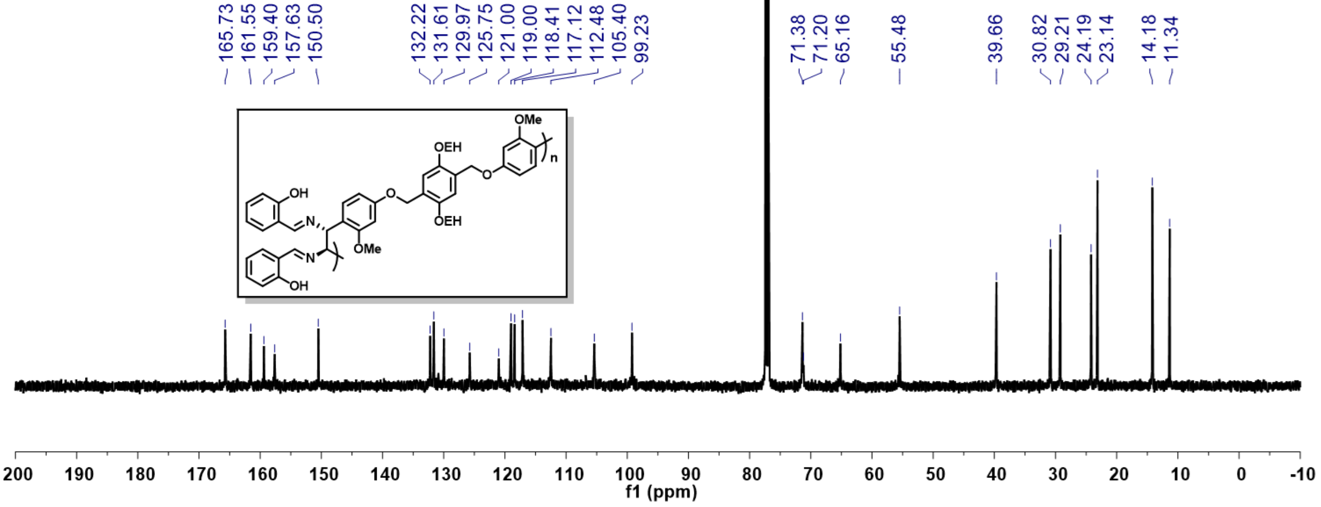

(*R*,*R*)-P3 ^{1}H NMR (400 MHz, $CDCl_3$)

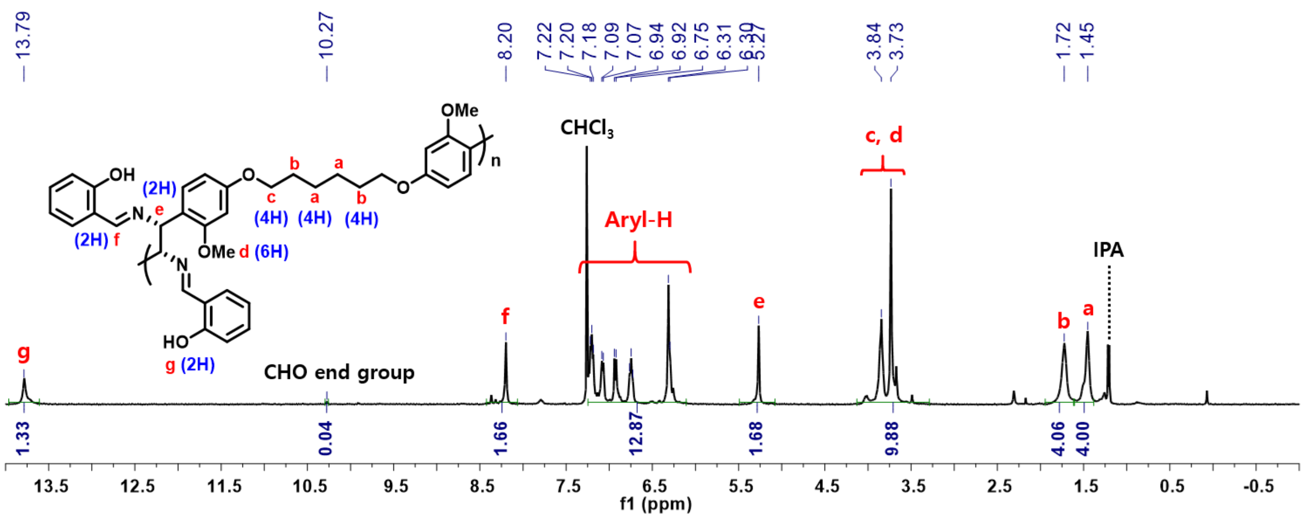

(*R*,*R*)-P3 ^{13}C NMR (125 MHz, $CDCl_3$)

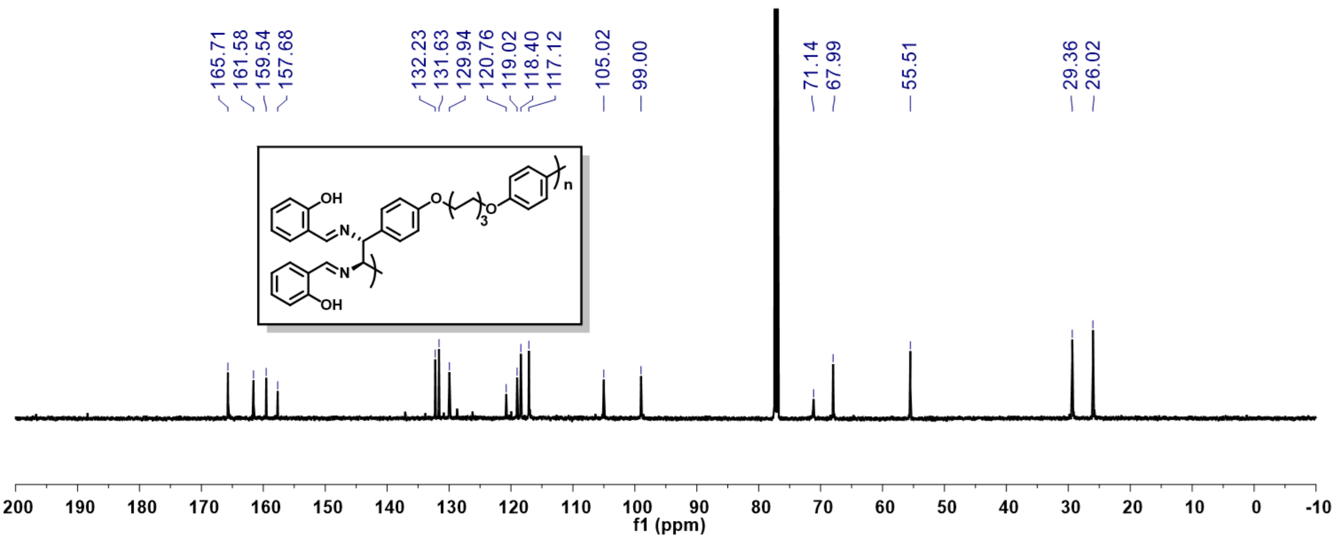

P4 ^{1}H NMR (400 MHz, $CDCl_3$)

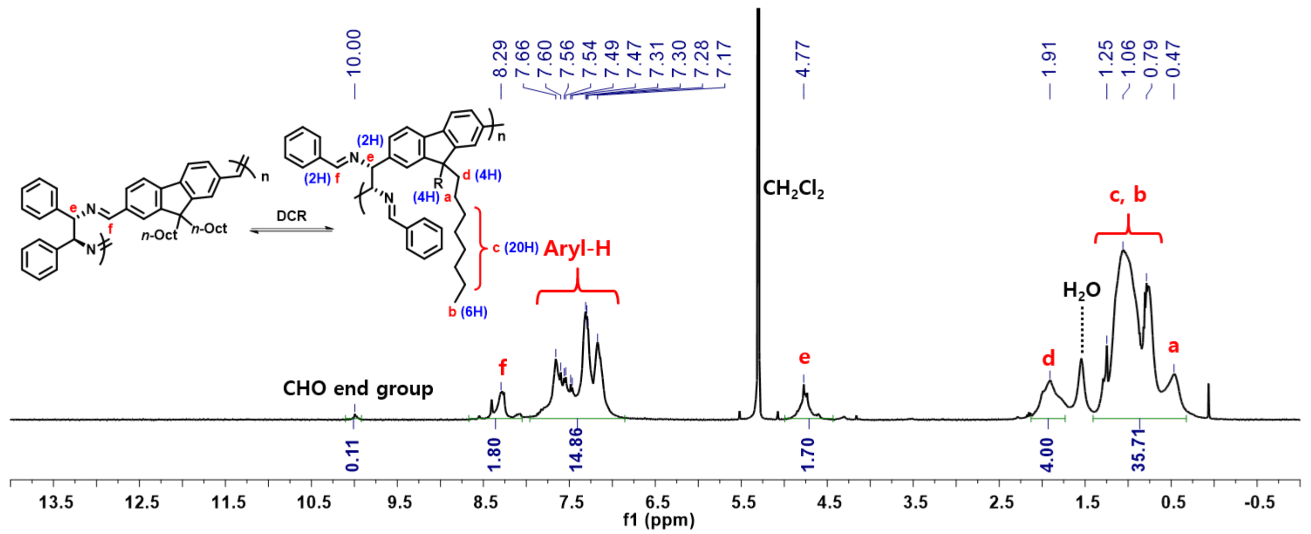

XI. References

1 Y.-J. Jang, S.-H. Hwang, T.-L. Choi. *Macromolecules* **2018**, *51*, 7476–7482.

2 G. Iadevaia, A. E. Stross, A. Neumann, C. A. Hunter. *Chem. Sci.* **2016**, *7*, 1760–1767.

3 M. Dickmeis, H. Cinar, H. Ritter. *Angew. Chem. Int. Ed.* **2012**, *51*, 3957–3959.

4 F. Vögtle, E. Goldschmitt. *Chem. Ber.* **1976**, *109*, 1–40.

5 M. Kim, H. Kim, H. Kim, J. Chin. *J. Org. Chem.* **2017**, *82*, 12050–12058.
